# The role of event understanding in guiding attentional selection in real-world scenes: The Scene Perception & Event Comprehension Theory (SPECT)

**DOI:** 10.3758/s13414-026-03234-7

**Published:** 2026-03-11

**Authors:** Lester C. Loschky, Maverick E. Smith, Prasanth Chandran, John P. Hutson, Tim J. Smith, Joseph P. Magliano

**Affiliations:** 1 Department of Psychological Sciences, Kansas State University, Manhattan, KS USA; 2 Present Address: Psychology and Counseling Department, Truman State University, Kirksville, MO USA; 3 Educational Testing Service, Princeton, NJ USA; 4 Creative Computing Institute, University of the Arts London, London, UK; 5 Department of Learning Sciences, Georgia State University, Atlanta, GA USA

**Keywords:** Scene perception, Event perception, Narrative comprehension, Film, Attentional selection, Eye movements

## Abstract

Your understanding of what you see now surely influences what you will look at next. Yet this simple concept has only recently begun to be systematically studied and elaborated within theoretical frameworks. The Scene Perception & Event Comprehension Theory (SPECT) distinguishes between front-end and back-end processes that occur while viewers perceive and comprehend dynamic real-world events. Front-end processes occur during each eye fixation (information extraction, attentional selection) and back-end processes occur in memory (the current event model, the stored event model, prior knowledge, and executive processes). We begin with a selective review of the scene perception literature on bottom-up and top-down effects on attentional selection in scenes, and highlight unanswered questions regarding the impact of the viewer’s event model–their understanding of what is happening now. Then, we outline the SPECT theoretical framework, and review empirical evidence about how the viewer’s current event model influences attentional selection. This influence is contrasted with those of visual saliency (e.g., color, brightness, motion) and task-driven control (i.e., goal setting, attentional control, inhibition). From this review, we specify a hierarchy of factors affecting attentional selection, in the order of task-driven control, visual saliency, and event models. We then propose several mechanisms by which the viewer’s event model influences attentional selection, and propose a systematic approach to investigating how that happens while watching dynamic scenes.

## Comprehension and attention in events

As you look around at the world, your understanding of what you see now influences what you will look at next. What you pay attention to greatly impacts your consciousness (Mack & Rock, 1998; Simons & Chabris, 1999), understanding (Just & Carpenter, 1987; Rouinfar et al., 2014), and memory (Tatler et al., 2005; C. C. Williams et al., 2005) for what you see. This raises the question, why do we attend to some things, but ignore others? We argue that our understanding of events influences what we attend to. Events are considered an important unit of cognition (Radvansky & Zacks, 2014). We intuitively view our experiences while engaging with the world as *events*. Events are typically defined as segments in time, at a location, perceived by a viewer to have a beginning and ending, with state changes in between (Zacks & Tversky, 2001). Importantly, events have components, such as agents, their behaviors, and cause and effect, within a spatiotemporal framework. Events are important to psychologists who study event cognition (Radvansky & Zacks, 2014), psycholinguistics (Altmann & Ekves, 2019), and narrative comprehension (Gernsbacher, 1990; Graesser et al., 1994; Trabasso et al., 1989). Psychologists who study how attention affects scene perception *are studying event cognition*, even if they may not construe that they are doing so; the colloquial definition of a “scene” is where something *happens*. As such, we contend that the study of attention and scene perception should explicitly embrace the construct of events, be informed of how they are represented, and study how and when those representations affect attention as events unfold dynamically. However, scene perception researchers have typically studied single events, at single times, using single static scenes (for reviews, see Epstein & Baker, 2019; Henderson & Hollingworth, 1999).

**Fig. 1:**
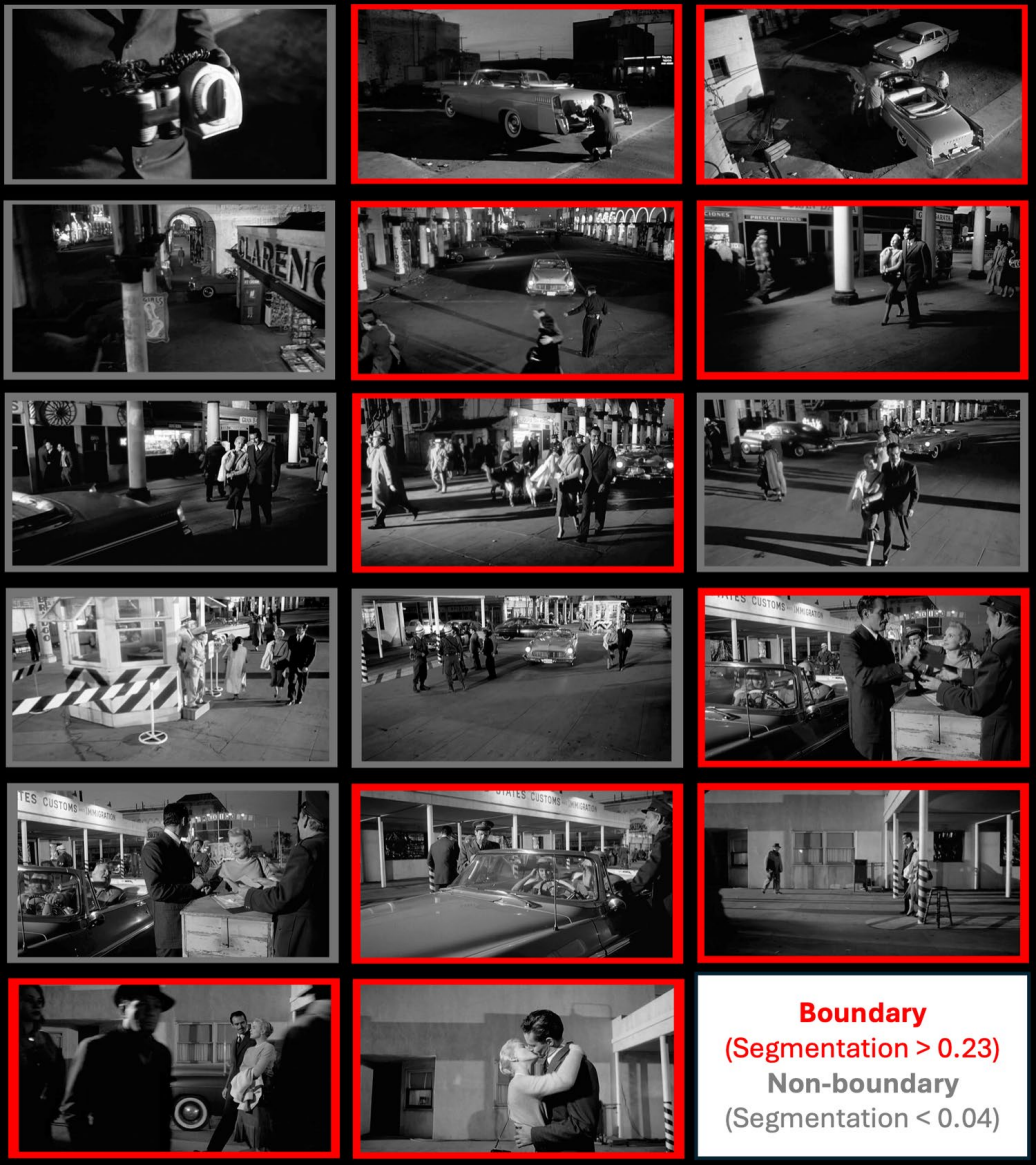
Frames illustrating taken from the film *Touch of Evil* (Welles & Zugsmith, 1958). Red borders around select frames illustrate normative event boundaries. These moments were judged as event boundaries by more than 23% of the participants. Gray borders indicate non-boundaries (judged as event boundaries by less than 4% of participants). (Color figure online)

To illustrate the importance of events for understanding attentional selection in scene perception, consider Fig. 1, which shows 17 frames from the opening scene of Orson Welles’ (Welles & Zugsmith, 1958) film *Touch of Evil*. In the first two events (shown with red borders), we see someone put a time bomb in a car, and soon after, the owners of that car unwittingly get in and drive off. In the third event (again with red borders), we see the car being stopped by a traffic cop on a busy street. The fourth event introduces another couple walking down the street. The walking couple then become the focus of the scene, while the bomb-laden car comes into and out of view over time. This creates heightened suspense in the viewer. Importantly, this suspense depends on the viewer’s understanding of the events they are seeing—their *event model*—because the bomb (a known entity) has the causal power to kill both couples (other known entities) at any moment. Does the viewer’s event model prioritize looking at the car whenever it enters the screen, even when the car is in competition with the walking couple for the viewer’s attention? Our answer to this question is a qualified yes, but as we will see, there are important constraints on how viewers’ event models influence their attention. Furthermore, individual frames in this scene, which would be treated as separate pictures in a scene perception study, have importance because of their relations to the viewer’s event model for the entire scene. Thus, we will use the *viewer’s event model* to explain how this scene is perceived and *how attention is deployed* as the scene unfolds. Importantly, under the right conditions, we get a significant and meaningful impact of viewers’ event models on their selective attention, as measured by where they send their eyes, including their first saccade on a scene (Hutson et al., 2022).

It is well-established that 1) what we selectively attend to greatly affects how we understand what we see; however, our example in the *Touch of Evil* scene suggests that 2) how we understand what we see affects what we selectively attend to. Concerning Point 1, there is ample evidence that attentional selection is essential to comprehension (for reviews, see Cohn & Foulsham, 2020; Foulsham et al., 2016; Just & Carpenter, 1987; Rayner, 1998; Rensink et al., 1997; Rouinfar et al., 2014). However, far fewer studies have investigated Point 2—how comprehension affects what we selectively attend to (Hutson et al., 2018, 2022; Loschky et al., 2015; Tanenhaus et al., 1995).

To capture the dynamic nature of real-world scenes, psychologists must study events that unfold over time. Much of our research examines the relationship between event comprehension and attentional selection by using narrative film stimuli (Hutson et al., 2017, 2022; Loschky et al., 2015). Visual narratives are an excellent type of stimuli to use for this purpose, because viewers process unscripted videos of everyday events and visual narratives very similarly (Magliano et al., 2020; Zacks et al., 2009). Put differently, movies work the way they do because they build upon the mechanisms that humans have developed for perceiving and understanding real-world scenes (Cutting, 2021; Zacks, 2015). Visual narratives, in virtual reality, film, slide shows, comics, or picture stories, capture the temporal evolution of events. People understand events using working memory and episodic memory representations called *event models* (e.g., Radvansky & Zacks, 2011). An event model in working memory reflects one’s understanding of the events as they experience them in real time. We argue that psychologists need to explicitly connect *attention*, in the context of dynamically unfolding scenes, to *event comprehension*, as reflected in the *event model*. To this end, we have proposed and begun testing the Scene Perception & Event Comprehension Theory (SPECT), which provides a theoretical framework for explaining how attentional selection and event comprehension are coordinated, as we experience events over time, in both unscripted dynamic real-world scenes and visual narratives (Loschky et al., 2018, 2020).

While Mary Peterson’s research program has not explicitly explored the top-down effects of event models on attention, our interest in developing a theoretical account of how that happens is in the spirit of her work. A key contribution of Mary Peterson’s work to the study of perception has been by looking for top-down effects on perception in an established research area where nobody thought to look for such effects. For example, even before her groundbreaking figure–ground work, Hochberg and Peterson (1987) showed that object recognition could unfold piecemeal over time, with partial perceptual information activating long-term memory representations that guide ongoing perceptual organization. This provided evidence against a strictly sequential model (first perception, then recognition) and instead supported a more interactive, real-time view of perception. Later, Peterson extended this line of inquiry to show that matching a proto-object to a long-term memory representation influenced the strongly assumed earlier process of figure–ground segmentation (e.g., M. A. Peterson, 1994; M. A. Peterson & Gibson, 1994). That was a theory-driven question, which called into question key assumptions about top-down effects on object processing. Our research on the role of event models on attention (e.g., Hutson et al., 2018, 2022) and perception (M. E. Smith & Loschky, 2019) is similarly one that few have explored. In this case, it is not that the influence of online event understanding on attentional selection seems counterintuitive—on the contrary, it appears quite intuitive to most people. Instead, it seems more likely that attention researchers consider the top-down theoretical construct of the viewer’s “event model” (i.e., their understanding of what they are seeing now) as too complex to test its influence on attentional selection. Research on visual attentional selection has long been largely limited to studies of visual search, often for very simple targets (e.g., conjunctions of red and green vertical and horizontal bars; or digits and letters created by removing segments of a figure-eight premask; e.g., Theeuwes et al., 1999; Wolfe, 2007). Only recently have attention researchers begun to study viewers’ eye movements while they watch real-world video clips, usually when studying the impact of visual saliency (as measured by a computational algorithm), or a task (e.g., search for a specific object in a scene; e.g., Henderson et al., 2007; T. J. Smith & Mital, 2013). Thus, our theoretically driven studies of how one’s event model influences attentional selection, may seem to “come out of left field” analogously to Mary Peterson’s studies of how object recognition influences figure–ground segmentation.

The goal of this paper is to discuss how viewers’ understanding of events, unfolding over time, influences their attentional selection. We focus on eye movements, rather than other attentional measures, such as self-paced dwell time in slideshows (Hard et al., 2011), because they provide a continuous and ecologically valid measure of attentional selection, across both time and space, as events dynamically unfold. We will first discuss what has been learned about attentional selection and event comprehension, either directly or incidentally, from prior research on scene perception. We will highlight gaps in this literature that necessitate the more targeted inclusion of *event models*. We then review SPECT (Loschky et al., 2018, 2020). In this review we add a well-established mechanism to SPECT, biased competition, to explain *a newly hypothesized mechanism for influencing attentional selection*, *the event model*. We then discuss recent research which is consistent with the event model hypothesis. Finally, we will discuss conclusions we can draw from our review of the research, unanswered questions raised by SPECT, and future directions for studying the effects of the event model on attentional selection in real-world scenes.

## What influences attention in real-world scene perception, and what important unanswered questions involve *event models*?

Prior work in scene perception research has investigated both bottom-up and top-down influences on attentional selection. Regarding top-down effects, this work has been limited to the effects of *tasks* and *prior knowledge*. However, to understand how viewers comprehend what they are seeing, from moment to moment, we need to invoke the theoretical construct of *event models*. The SPECT theoretical framework was created, in part, to explain how event models influence attentional selection. Below is a selective and condensed overview of studies of attentional selection in real-world scenes. In reviewing these studies, we make the case for *why events* are critical for understanding attentional selection in scene perception.

### Effects of bottom-up visual saliency on attentional selection in scenes

**Fig. 2:**
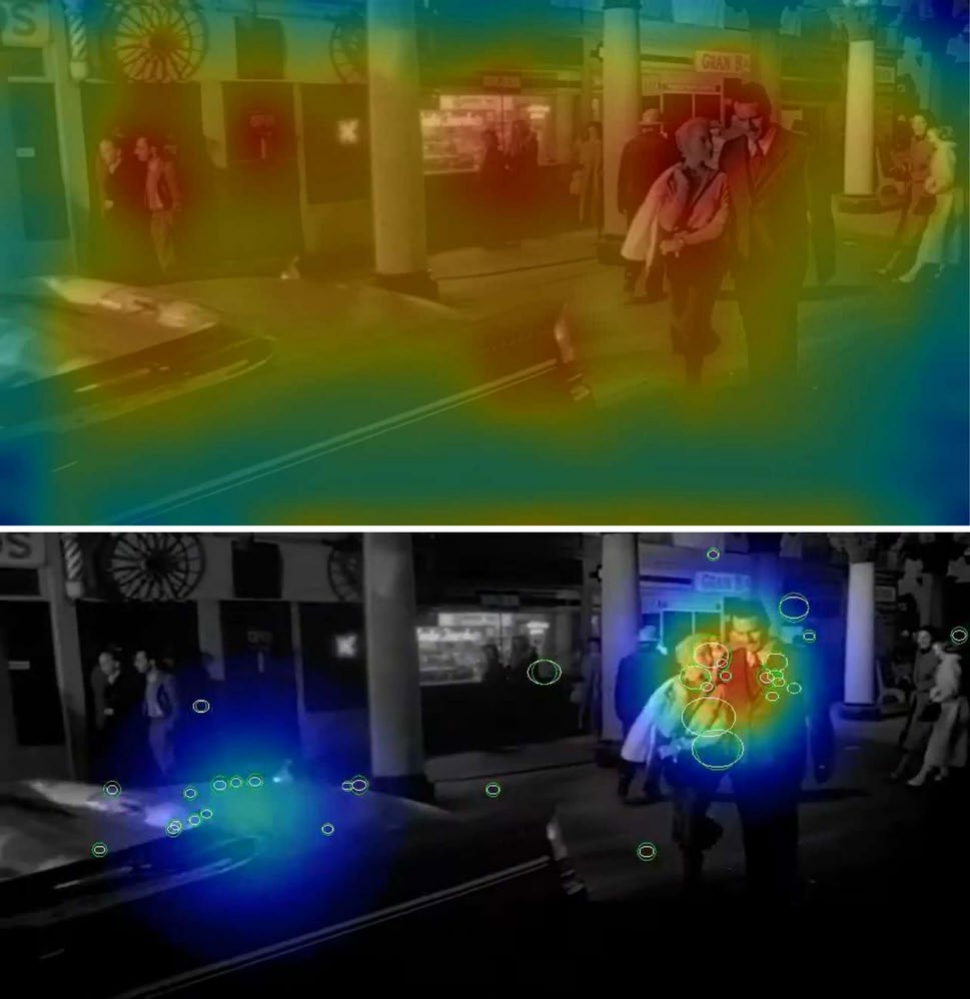
Top: Saliency map (Linardos et al., 2021) of a frame from the opening scene of Orson Welles’ *Touch of Evil* (Welles & Zugsmith, 1958). Bottom: Fixations and heat map on that frame from *Touch of Evil*. Note the low predicted saliency of the car trunk, compared with observed fixations on the car trunk, which these viewers know contains a bomb (data from Hutson et al., 2017). (Color figure online)

Bottom-up visual stimulus features and top-down factors influence attentional selection. Features that predict where people look are termed *visually salient*. Computational models of visual saliency have been developed and studied for roughly 30 years (Bruce & Tsotsos, 2009; Itti & Koch, 2000; Kummerer et al., 2017; Linardos et al., 2021; Tsotsos et al., 1995). Such models generate a 2-D saliency map, indicating the predicted probability of a viewer’s attentional selection in an image. Early models used low-level bottom-up visual feature contrast (e.g., luminance, color, orientation, and motion) to predict saliency (e.g., something light among dark things, something red among green things, something vertical among horizontal things; Itti & Koch, 2000). The most visually salient features are things that suddenly appear (Theeuwes et al., 1999), or are moving (Carmi & Itti, 2006; Mital et al., 2010). More recent computational saliency models have included higher-level semantic object classes known to strongly attract human gaze, such as human faces (Fletcher-Watson et al., 2008), or text (Cerf et al., 2009), to predict viewers’ attention (Kummerer et al., 2017; Linardos et al., 2021). Figure 2 shows an example of the predicted saliency of regions in a screenshot from the film, *Touch of Evil*. Computational models perform above chance at predicting attentional selection; however, even the best models cannot explain significant proportions of variance in where people look, because top-down factors also influence attentional selection, especially in static scenes (Foulsham & Underwood, 2007; Henderson et al., 2007; Pedziwiatr et al., 2021; Tatler et al., 2011). As shown in the top of Fig. 2, a saliency map based on high-level features (Linardos et al., 2021) predicts that the walking couple are the most salient region of that film frame, whereas the trunk of the car carrying the bomb is not. The bottom of Fig. 2 shows a heat map based on eye fixations, from viewers who knew of the bomb in the car trunk, and maintained that knowledge in working memory throughout the 3-min scene. Although viewers’ eye fixations were consistent with the high-level saliency model’s predicted *hot spot* on the walking couple, the saliency model did not predict the secondary hot spot on the car trunk. Conversely, the hot spot on the car trunk was both predicted, and shown, to be important for those viewers whose event models included the concealed bomb (Hutson et al., 2017, Exp. 2 A).

A key unanswered question is how bottom-up visual saliency and a top-down event model biases compete for attentional selection in ongoing scenes, such as visual narratives, and dynamic real-world scenes. The salience, the strength of bias from an event model, and the balance between the two are likely to change as a scene unfolds over time.

### Effects of task-driven top-down attentional selection in scenes

We distinguish between two types of top-down influences on attentional selection, which we will call “*task-driven*” versus “*knowledge-based*” (see also Baluch & Itti, 2011). Task-driven top-down influences on attentional selection operate in everyday tasks like searching for your car in a parking lot. Computational models of visual search have become increasingly accurate at predicting where people will look while searching for targets (e.g., a red crossover SUV in a parking lot full of cars of various colors, types, and models; Wolfe, 2021; Zelinsky et al., 2020). Such visual search models use a 2-D priority map (like a saliency map, but including goals) to indicate the predicted probability that a viewer will fixate each image location. Importantly, a viewer’s task is a much better predictor of what they will attend to in a scene than visual saliency (Einhäuser et al., 2008; Foulsham & Underwood, 2007; Henderson et al., 2007; Tatler et al., 2011) even in real-world scene videos (T. J. Smith & Mital, 2013), despite the fact that *motion* creates the strongest form of visual saliency (Mital et al., 2010).

A key unanswered question is the balance between *task-driven*, *knowledge-driven*, and specifically *event model-driven biases* in the competition for attentional selection. In contrast to task-driven situations, many everyday experiences, such as watching a movie or reading a graphic novel, unfold without an explicit external goal. In these cases, event models may guide attentional selection by default, organizing perception and memory in service of the internal goal of enjoyment (Vorderer et al., 2006). Such enjoyment is a rewarding state that emerges from the dynamic interaction between viewer motives and media features (e.g., narrative coherence, pacing)

### Effects of knowledge-driven top-down attentional selection in scenes

#### **Gist and schemas guide visual search in scenes**

Studies have investigated knowledge-driven top-down influences on viewers’ attentional selection in real-world scenes based on the viewer’s relevant *schemas* (i.e., structured, generic, semantic long-term memory representations). Viewers can rapidly extract a holistic semantic representation of a scene within their first fixation (e.g., ≤330 ms); commonly known as its *scene gist* (Greene & Oliva, 2009; Larson et al., 2014; Loschky et al., 2007; Oliva & Torralba, 2006). The scene’s gist activates its superordinate and basic-level category, the schema of the scene in semantic memory, and relevant knowledge about what objects tend to be where (Võ et al., 2019). It can also include the basic level category of one or more key animate entities in the scene (e.g., a man, a dog; Fei-Fei et al., 2007), the identity of an agent (i.e., the “doer” of an action; Hafri et al., 2013), and the scene’s emotional valence (e.g., neutral, scary, happy; Calvo & Lang, 2005).

A key finding is that because a scene’s gist rapidly activates a viewer’s schemas in semantic long-term memory, it influences attentional selection of their first eye movement (saccade) on a scene. For example, Eckstein, et al. (2006) found that, when searching for a chimney, viewers’ first saccade went to scene locations where chimneys were expected to be (i.e., the roof of a house). This was true, even when no chimney was there (Eckstein et al., 2006; Torralba et al., 2006). This is evidence that a schema (e.g., knowledge that houses have chimneys on their roofs) influences attentional selection (i.e., where to send a saccade). Note that this example combines task-driven top-down attentional selection (due to carrying out an explicit search task) and knowledge-based attentional selection (due to activating a relevant schema influencing saccade target selection). Note also that the speed of influencing attentional selection—namely, the first saccade on the image, was likely due to having time to process the search target identity (i.e., the word “chimney”), and activate its scene schema, before seeing the scene image.

More recently, Henderson and colleagues have extended this work by examining how the distribution of local scene meaning affects attentional selection (Hayes & Henderson, 2022b; Henderson & Hayes, 2018; Peacock et al., 2019). They obtained a 2D spatial distribution of local scene meaning comparable with a bottom-up visual salience map. For that, they generated meaning maps by crowdsourcing participants’ ratings of the meaningfulness of many isolated patches taken from scenes. Henderson and colleagues have consistently demonstrated that meaning maps account for unique variance in the distribution of attention, even after statistically controlling for the correlation between meaning and bottom-up saliency. Such knowledge-based attentional guidance occurs because the scene’s gist accesses stored knowledge about the scene category and a coarse representation of where informative regions in a scene are likely to be. However, the precise information that meaning maps capture remains an open debate (Hayes & Henderson, 2022a; Pedziwiatr et al., 2021).

A key unanswered question is how the scene gist, acquired on the first fixation of a scene, is used to *lay the foundation* for the *current event model*, *an integrated dynamic meaning representation,* and how that, in turn, influences attentional selection. Gist activates schemas from semantic long term memory which contribute to the starting point (i.e., foundation) for the current event model, as described in detail later.

#### **Effects of schema-inconsistent objects on attention in scenes**

What happens to viewers’ attentional selection in a scene if an object violates the viewer’s schema for that scene? For example, imagine a bathroom scene in which one sees a tube of toothpaste versus a small flashlight on a bathroom sink (Coco et al., 2020). Will the difference in the viewer’s expectations for each object in the bathroom scene produce differences in attentional selection? Research has gone back and forth on this question, but it appears that the following statements are true: 1) viewers can detect a schema-inconsistent object using their parafoveal vision on *the last fixation before* they first fixate such an unexpected object (Coco et al., 2020); 2) once the viewer fixates such an unexpected object, their fixation durations are longer, and they may make extra fixations in an attempt to integrate the unexpected, schema-inconsistent object into their scene representation (which we would stipulate is their *event model* of the scene; Coco et al., 2020; Cornelissen & Võ, 2017; De Graef et al., 1990; Gareze & Findlay, 2007; Henderson et al., 1999; Võ & Henderson, 2011); 3) highly salient objects tend to be fixated quickly, regardless of their scene schema consistency, which can eliminate the top-down effect of schema-inconsistency (Underwood & Foulsham, 2006).

Do such schemas play a role in guiding attention when the scene is dynamically changing, such as in movies, or in dynamic real-world scene videos? The assumption is that prior schema findings should apply to studies using dynamic scenes. However, by definition a schema can only influence our behavior if we believe it to be valid within a scene. It is currently unknown how schemas and scripts emerge or influence behavior in dynamically changing scenes. The concepts of schemas and scripts may be too limiting a theoretical construct to achieve that goal. *Event models*, which incorporate schemas, but are *specific to the given situation at that moment*, are needed to achieve greater breadth of explanatory power in terms of understanding what is expected, and how that affects attentional selection. For example, in Fig. 1, our gist of parking lots and street scenes activate those schemas, which include cars; however, as the scene continuously unfolds, *the particular car* driven by the first couple is expected to appear and reappear, because it is represented in the viewer’s *current event model*. Moreover, the car’s significance to the scene is based on the viewer’s event model *including the presence of the bomb in its trunk*, which is known, but not seen.

Additionally, the well-replicated finding of more fixations on scene-inconsistent objects, once they are found, is sometimes attributed to difficulty in “integrat[ion] with the scene representation” (Coco et al., 2020; Henderson et al., 1999, p. 226). However, little has been said about the nature of such *integration* processes. What is needed, then, is a theoretical framework that contains integration processes, including their inputs and outputs, what happens when integration processes fail, and how they affect attentional selection. In SPECT, such integration processes are handled by the *mapping* process of the current event model.

#### **Studies exploring the relationship between event models and attentional selection**

Some prior research has considered events, either explicitly or implicitly when studying attentional selection. These studies were conducted to show how task instructions affect comprehension, but in the context of pictures and tasks that afford the creation of an event model (Borji & Itti, 2014; DeAngelus & Pelz, 2009; Kaakinen et al., 2011; Lahnakoski et al., 2014; Yarbus, 1967). However, it is important to note that the researchers did *not* describe their studies in terms of event models, thus we reinterpret them as such here.

Yarbus (1967) is recognized as a seminal study demonstrating “top-down” or “higher-level” influences on attentional selection. While this is certainly the case, we argue that it is also a seminal study demonstrating that *event models* affect attentional selection. Yarbus asked a participant several questions about a painting while their eyes were tracked. The painting showed a man entering a living room where several family members were watching him. Several of the questions required the participant to draw *inferences*, such as determining the relative wealth of the family members, estimating their ages, and what they had been doing before the visitor arrived. We argue that *inferences* evoked by the questions fostered the development of an *event model* for the picture because they emphasized situational information that could not be derived solely from the visual content. In Yarbus’ demonstration, the viewer’s eye movements differed considerably between questions, indicating that their attentional selection was affected by the inferential processes. Later studies have replicated and extended these results with more participants (DeAngelus & Pelz, 2009) and more stimuli, while controlling for visual saliency (Borji & Itti, 2014; but see Greene et al., 2012).

Other studies have investigated how viewers’ event models influenced their attentional selection in scenes by manipulating viewers’ *cognitive perspective*. Here the theoretical construct of the event model is more extensively implicated, though not always explicitly discussed. Kaakinen et al. (2011) had participants view photographs of home interiors while imagining themselves as either a burglar or a home buyer (originally used in Anderson & Pichert, 1978). They found that adopting different perspectives guided viewers’ attentional selection to objects relevant to each—for example, for the burglar perspective, a handbag or a painting, versus for the home buyer perspective, a discolored toilet seat or a wooden door. Similarly, Lahnakoski et al. (2014), manipulated viewers’ imaginary perspective while they watched a video clip from an episode of *Desperate Housewives.* Participants alternatively adopted the perspectives of either a detective or an interior decorator. For the detective perspective, key information came from the two characters, usually near the center of the screen, while for the decorator perspective, key information came from the background, usually in the periphery of the screen. Viewers’ fixation locations in the two perspectives differed qualitatively, but not significantly above chance. Whether the visual stimulus affords event model expression via viewer eye movements is an important factor. Static scenes impose fewer temporal demands on attention than dynamic or edited film sequences, which continually change. Because Lahnakoski et al. (2014) used edited TV clips composed by filmmakers to guide viewers’ attention, this may have minimized the scope for viewers to use their event models to deviate from the filmmakers’ intended viewing patterns. Likewise, although Kaakinen et al. (2011) used still images (which as noted above have less saliency-engendering features than movie clips), they found that the effect size of visual saliency was larger than that of cognitive perspective (which we construe as involving the viewers’ event models).

As noted above, most of these studies investigated “knowledge-driven” top-down effects, but did so by giving participants an explicit task. That is good experimental practice, since otherwise, participants may not know what to do (Henderson et al., 2007). Thus, we assert that the effects shown are a combination of task-driven attentional selection, via the executive process of goal-setting, knowledge-driven attentional selection, based on activating schemas in semantic long-term memory, and using that activated prior knowledge to create event models.

A key unanswered question is the degree to which the top-down theoretical construct of the *viewer’s event model* influences their attentional selection *in the absence of an explicit task goal*—understanding a visual narrative is something viewers spontaneously do by default. Future research should determine the extent to which visual saliency and the event model determine viewers’ attentional selection, both in static events (e.g., slide shows, picture stories, or comics) and dynamic events (e.g., real-world videos, movies, virtual reality).

## The Scene Perception & Event Comprehension Theory (SPECT)

**Fig. 3:**
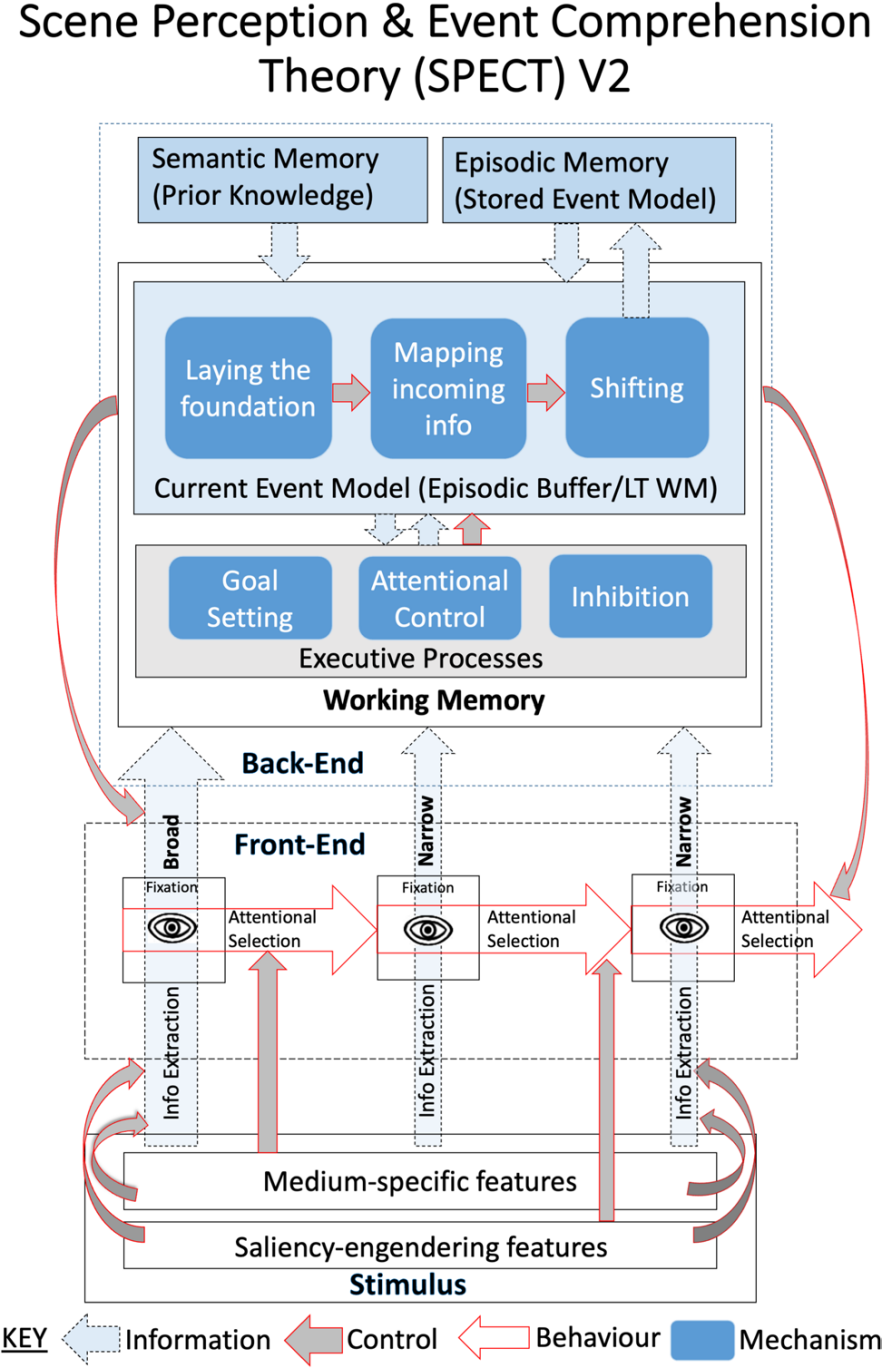
The SPECT box and arrow model revised based upon the earlier version (Loschky et al., 2018, 2020) for clarity. (The Appendix at the end of the paper describes the two substantive changes, changes for greater comprehensibility, and changes for greater clarity.) (Color figure online)

SPECT provides a theoretical framework for explaining how a viewer’s event model influences what they selectively attend to in real-world scenes and events. SPECT is unique in having such a comprehensive account of how high-level cognitive constructs, specifically within event models, affect something that occurs during every eye fixation, attentional selection. Before SPECT, theories of attentional selection have not included such higher-level cognitive constructs as the event model. Likewise, before SPECT, theories of real-world event comprehension have not explained how higher-level comprehension processes might influence lower-level constructs like attentional selection (Loschky et al., 2018, 2020). As shown in Fig. 3, SPECT distinguishes between stimulus features, front-end processes, and back-end processes. We describe each of these, and their sub-parts, below. We also note that Fig. 3 is a revised version of the previous SPECT box and arrow model (Loschky et al., 2018, 2020), which has primarily been edited for clarity. (The Appendix at the end of the paper describes the two substantive changes, changes for greater comprehensibility, and those changes for greater clarity.)

### The stimulus

The starting point for SPECT is the visual stimulus. The stimulus can be composed of either static or dynamic real-world scenes, and can vary in complexity and realism. Stimulus properties constrain front- and back-end processes because they influence attentional selection. SPECT assumes that there are two aspects of the stimulus that affect attentional selection, saliency-engendering image features, and medium-specific features.

#### Saliency-engendering stimulus features

SPECT assumes that *most* saliency-engendering visual features operate similarly across different visual media. However, there is at least one medium-*specific* visual feature—namely, those changes across film frames that produce *motion* in film, video, and virtual reality—which is absent in still image media (i.e., comics, slideshows, or picture stories). Computed motion has a large impact on attentional selection (Carmi & Itti, 2006; Mital et al., 2010). Critically for SPECT, computing image salience requires no prior knowledge of the people in the scene, their interactions, or of what came before the current scene. Thus, in the saliency map shown in Fig. 2, from the film *Touch of Evil*, the saliency algorithm does not predict the car trunk to be salient. Conversely, if the viewer’s *event model* contains the information that *there is a bomb in the car trunk*, that region becomes highly important and more likely to be attended, as shown in the bottom of Fig. 2. Note, however, that here we are only concerned with the saliency-engendering *features in the stimulus*—the actual computation of saliency is described as a sub-process of *attentional selection* in the *front-end* of SPECT.

#### Medium-specific features

Medium-specific features are aspects of visual design that can influence attention. These include individual pictures in wordless picture stories, the organization of panels and text in comics, and the shots, cinematography, and spatial arrangements of characters and objects in films (T. J. Smith, 2013). For example, narrative films follow conventions of editing (Bordwell & Thompson, 2003) and other stylistic features (Hinde et al., 2018). Studies have demonstrated their impacts on eye movements (T. J. Smith & Martin-Portugues Santacreu, 2017), covert attention (Hinde et al., 2018), event perception (Cutting & Iricinschi, 2015), and affective responses (Lankhuizen et al., 2022). Such design features may guide viewers’ attention to the most relevant visual information in a film shot or comic frame (Cohn, 2020; T. J. Smith, 2013). As illustrated in Fig. 1, conventions of panel structure inform the order in which panels are attended. Comics written in English, for example, are typically read in a “Z” pattern (i.e., left to right + top to bottom), though additional panel-specific constraints also affect reading order (Cohn, 2013). Some design features must be learned, such as the Z-pattern, whereas others do not. For example, after a *cut between shots* in a film (i.e., continuous camera run), viewers naturally tend to fixate near the center of the screen (T. J. Smith, 2013; Wang et al., 2012).

### The front end

As shown in Fig. 3, the front end of SPECT has two components: 1) information extraction and 2) attentional selection. SPECT distinguishes between front-end processes that occur during every eye fixation, and back-end processes that occur in working memory and long-term memory. This distinction reflects the fact that only a limited portion of the visual field can be extracted during a single fixation, and minimal visual information is extracted during the saccades between fixations (Dorr & Bex, 2013; Ross et al., 2001). Once a fixation ends, the extracted information can only be processed further through passively activated long-term memories or more actively maintained short-term/working memories (Hollingworth, 2009; Irwin, 1996). Short-term/working memory is relatively abstract (Carlson-Radvansky & Irwin, 1995), and limited to only three to four objects or chunks (Irwin, 1996; Irwin & Gordon, 1998; Luck & Vogel, 1997). Thus, the distinction between the rich perceptual information available during fixations and the more limited preprocessed information used in memory is very important. Furthermore, attentional selection largely determines which elements of the available visual information are encoded and maintained in working memory (Irwin, 1996; Irwin & Gordon, 1998).

#### Information extraction

Figure 3 symbolizes the construct of information extraction in the front end with upward arrows from an eye icon to the back end, symbolizing passing extracted information to the event model in working memory. Information extraction encompasses everything that happens from the moment that light hits the retina until that activates the relevant semantic representations. Those include the identity of the scene, or the person(s), object(s), or event(s) within it. Current theories of object, scene, and event identification are strongly influenced by the success of feed-forward convolutional neural networks (CNNs) at these tasks, which currently rival human accuracy (Kriegeskorte, 2015). Furthermore, studies have shown notable similarities to processing done by such neural networks and the activity of cells and regions of the ventral visual stream. Those include the visual cortical areas V1, V2, V4, inferior temporal cortex (IT), and lateral occipital cortex (LOC) for objects (Cichy et al., 2016; Yamins et al., 2014). They also include the parahippocampal place area (PPA) and occipital place area (OPA) for scenes (Cichy et al., 2017; for review see Wardle & Baker, 2020). Importantly, however, studies have also shown the importance of recurrent processing, including feedback connections, which is widespread in primate visual cortex, but not included in purely bottom-up/feed-forward CNN models (Kietzmann et al., 2019; Wardle & Baker, 2020). As we will point out later, top-down feedback connections are important for SPECT, which argues that the back-end event model influences front-end information extraction (Smith, 2021; Smith & Loschky, 2019).

Information extracted on each eye fixation is fed to the back end to support event model construction. Information extraction is either broad, encompassing much of an image, or narrow, focusing on only a small part of the scene, such as a single object (Ringer et al., 2016; Seiple et al., 2002; L. J. Williams, 1988). Broad information extraction occurs primarily in peripheral vision, which is the vast majority of the human visual field (i.e., ±105° eccentricity, horizontally from the point of fixation). Peripheral vision (from >5° eccentricity) is important for rapidly identifying the gist of a scene within a single eye fixation (Larson & Loschky, 2009; Loschky et al., 2019). Conversely, narrow information extraction occurs in central vision (<5° from fixation), which, by itself, is less important for acquiring scene gist (Larson & Loschky, 2009). On the other hand, there appears to be a bias for the first 100 ms of the first eye fixation on a scene to spread outward from central vision to peripheral vision (Larson et al., 2014).

Research also suggests that viewers transition from a broad mode of information extraction to a narrow mode as they make eye movements (i.e., saccades) in a scene (Pannasch et al., 2008). Namely, the first 2–3 s of scene viewing are in the *ambient mode*, in which viewers’ *saccades are longer*, going further into the visual periphery, but their *fixation durations are shorter*, indicating broad information extraction. The ambient mode allows viewers to rapidly locate key information in the scene. Then, from 3–6 s of processing of a scene, viewers shift to the *focal mode*, in which their *saccades are shorter*, staying within parafoveal or foveal vision, but their *fixation durations are longer*, together indicating more detailed, narrow information extraction. The focal mode enables viewers to selectively extract detailed information from specific objects of interest. Viewers need approximately two fixations to extract narrower, more basic information, such as the details of a specific person or action in a scene (Larson, 2012). Increasingly detailed information is extracted on a fixation-by-fixation basis in the *front end*, and is accumulated across multiple fixations in the *back end*.

Importantly, front-end *information extraction* is influenced by the back-end *event model* processes. Specifically, having a spatiotemporally coherent event model in the back-end facilitates rapidly recognizing the gist of a scene in the front-end, as quickly as 100 ms after light hits the retina (M. E. Smith, 2021; M. E. Smith & Loschky, 2019). Conversely, there is a decrease in information extraction immediately before and after an event boundary (Crundall et al., 2002; Huff et al., 2012; Ji & Papafragou, 2022; Yates et al., 2024).

#### Attentional selection

Figure 3 illustrates the construct of attentional selection: rightward arrows between successive eye icons show that attentional selection mechanisms determine *what information* is processed during fixations, and *where to send the eyes* next. These two operations occur in parallel during each eye fixation (Findlay & Walker, 1999).

**Fig. 4:**
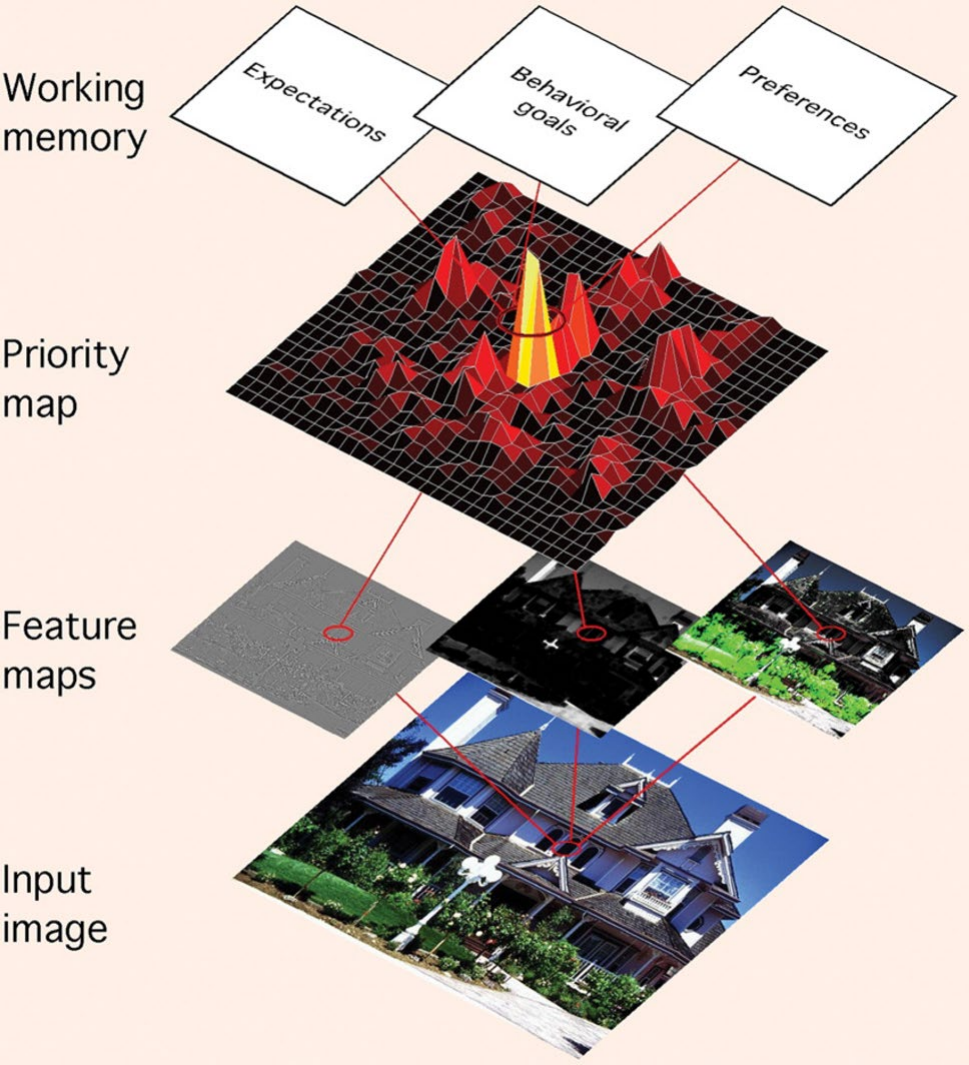
Graphic representation of the biased competition approach to attentional selection (Ptak, 2012). (Color figure online)

Here, as shown in Fig. 4 (Ptak, 2012), we update SPECT by incorporating fundamental concepts and assumptions from the *biased competition* approach to attentional selection. It is a widely supported approach, developed over the past 30 years (Adeli & Zelinsky, 2018; Desimone & Duncan, 1995; Ptak, 2012; Tsotsos et al., 1995). It draws on converging evidence from behavioral and neurophysiological research (Bichot et al., 2005; Buffalo et al., 2010; Desimone & Duncan, 1995), and computational modeling (Adeli & Zelinsky, 2018; Hamker, 2004; Itti & Koch, 2000; Tsotsos et al., 1995). Incorporating these important details to the SPECT theoretical framework opens avenues for future computational modeling efforts which integrate existing computational models of attentional selection. Critically, the biased competition approach captures how a viewer’s task, prior knowledge, and—as we argue here— *their event model*, *bias* bottom-up competition for attentional selection

As the name suggests, the approach includes bottom-up *competition* and top-down *bias*. Bottom-up competition is represented by a *saliency map;* top-down biases transform this saliency map into a *priority map.*

In describing the biased competition approach to attentional selection, we will refer to processing “units” within “layers,” consistent with computational models of object recognition discussed above. This is more abstract than “cells” within “brain areas.” But the abstraction is based on a relatively strong correspondence between *units in layers* and *cells in brain regions* as shown in computational neurophysiological studies (Nonaka et al., 2021; Yamins et al., 2014). In Fig. 4, each rectangular image represents a layer. In this architecture, bottom-up input ascends through multiple layers (e.g., hidden layers in a connectionist or deep neural network). Saliency and priority maps are implemented in higher layers, as shown in Fig. 4.

A key feature linking these maps is *retinotopic mapping*. This enables a spatial correspondence between regions in an input image, and in layers, as illustrated in Fig. 4. Retinotopic mapping enables selective attention to direct eye movements. The winning location in the priority map becomes the next fixation point.

SPECT captures the inherent strengths and limitations of peripheral vision by incorporating *cortical magnification of the fovea.* Namely, the fovea and parafovea (center of vision) cover only about 10° of the horizontal visual field, but have ~40% of the cells in area V1. This leaves the remaining 210° of the horizontal visual field^1^ to be processed by only the remaining 60% of the cells (Curcio et al., 1990; Daniel & Whitteridge, 1961). Therefore, two small objects in the fovea may each be represented by their own separate cortical cells, but in the periphery the same two objects may share a single cell. Such cases produce a jumbled neural representation, and thus perceptual confusion, called “crowding” (Strasburger & Malania, 2013). Most saccades are short to medium, with very few long ones, and this is explainable in terms of cortical magnification of the fovea (Raabe et al., 2023). Thus, models of attentional competition should incorporate cortical magnification, though only a few currently do (Adeli et al., 2017; Adeli & Zelinsky, 2018; Raabe et al., 2023).

SPECT also acknowledges the well-known divergence in visual processing between the “what” and the “where/how” pathways (for reviews, see Kravitz et al., 2011, 2013; Milner & Goodale, 2008). *Entities* (people, objects) are represented in the “what” pathway; whereas, the *locations* of those entities, and their *actions* are represented in the “where/how” pathway. Saliency and priority maps likely exist in both pathways (Fecteau & Munoz, 2006; Ptak, 2012; Zelinsky & Bisley, 2015).

Within each layer, *competition* selects the winning location for the next eye movement. The exact mechanisms of reducing the activity of most units, while amplifying ultimately one, vary between biased competition computational models (Adeli & Zelinsky, 2018; Hamker, 2004; Itti & Koch, 2000; Tsotsos et al., 1995). Regardless of the mechanism, the bottom-up/feed-forward sweep of activity combined with an inhibitory mechanism produces a “winner” within a retinotopic map. As shown in Fig. 4, such competition likely occurs in multiple feature maps, each for processing a different type of visual feature (e.g., color, luminance, orientation; Itti & Koch, 2000). Other high-level maps may also exist with winners being faces, or written text, both of which are known to strongly attract viewers’ gaze (Cerf et al., 2009). Winners from these maps compete in the final saliency map, where a single retinotopically mapped location is selected as most salient. This is shown in Fig. 4 as the central map having multiple peaks. But why is it labeled as a “priority map” instead of a “saliency map”?

The answer is that a winner based on saliency can be overridden by top-down *biases*, thus transforming what was the saliency map into a *priority map*. As shown in Fig. 4, top-down bias spreads through feedback connections that begin at the top layer and go down sequentially through lower layers (Adeli & Zelinsky, 2018; Hamker, 2004; Tsotsos et al., 1995). Again, the exact mechanisms of excitation and inhibition vary among computational models. Whether the top-down bias is strong enough to overcome bottom-up saliency determines the winner of the biased competition for each new fixation.

The top of Fig. 4 illustrates several well-documented sources of top-down bias. One is the viewer’s task/behavioral goals (e.g., find X). Another is their expectations from prior knowledge/schemas (e.g., places where X are often found). A third is learned preferences based on reward values (e.g., X are good, or Y are dangerous). But, as noted in Fig. 4, all are contained in *working memory*, which is privileged for visual attentional selection (Han & Kim, 2009; Olivers et al., 2006; Soto et al., 2005). Working memory can guide attention regardless of whether its contents are visual or verbal (Soto & Humphreys, 2007), and whether one or multiple objects are held in mind (Bahle et al., 2018).

Critically, SPECT proposes that the viewer’s *current event model* residing in working memory can exert such a top-down *bias* on attention. As we will discuss in the section entitled, "The current event model," the event model in working memory tracks entities (e.g., people, or objects), actions, locations, time, goals, and causal relations. Thus, the event model representation in working memory could create a top-down bias focusing on a given person, location, time, action, or goal-object, which in turn, could amplify the activity of relevant units corresponding to those event indices in the *what* or *where/how* pathways.

##### **The roles of task versus prior knowledge and event models in biased competition for attentional selection**

**Fig. 5:**
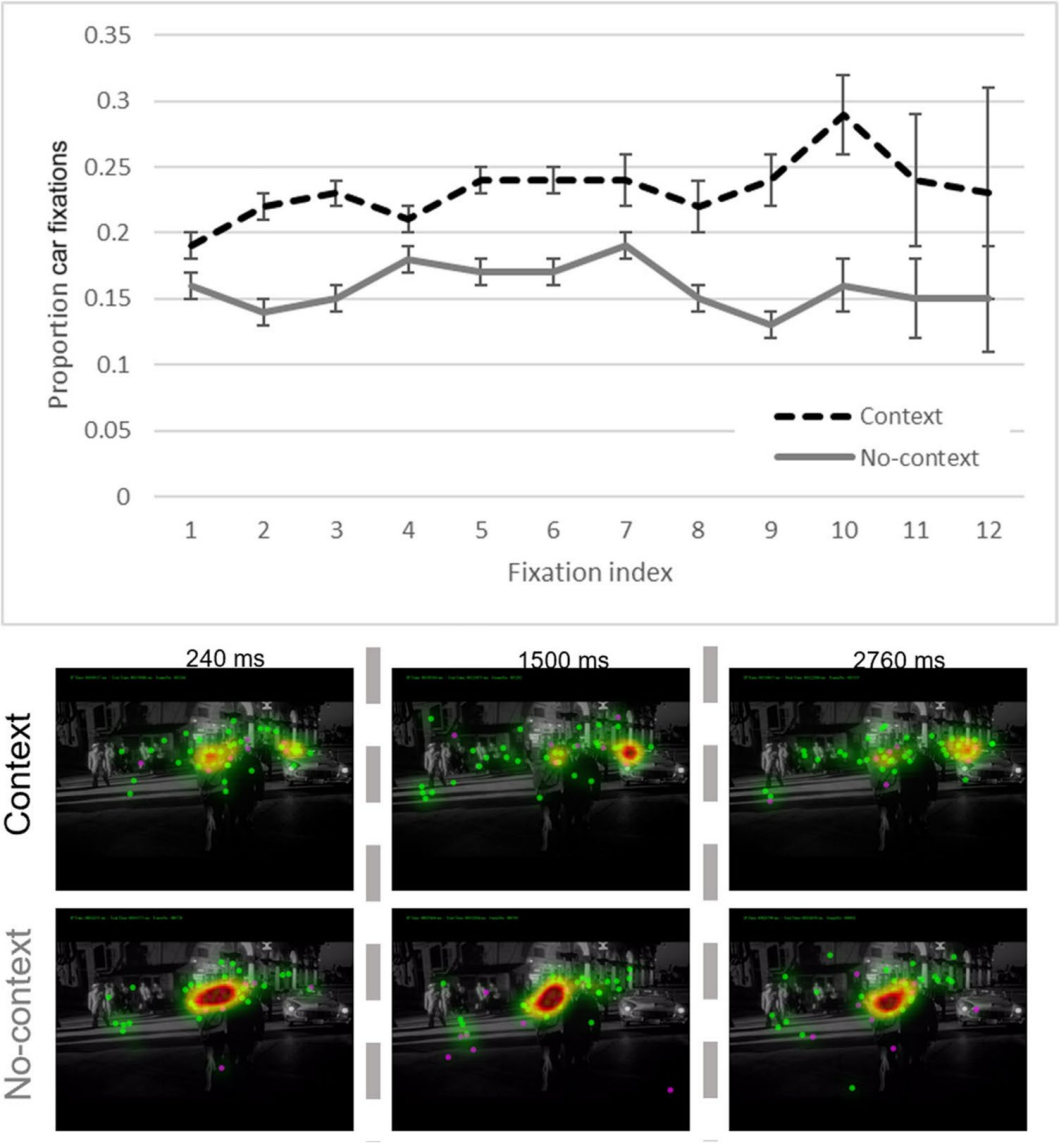
Above: Proportion of participants who fixated the car with the bomb for each fixation on a given slide. Context participants are in dashed black; no-context in solid gray. Error bars are standard errors, which increased with fixation index due to lower *N* with increasing numbers of fixations during the 3-s viewing period (i.e., not many participants made 11 or 12 fixations on a single slide). Below: Example heatmap images for the context and no-context conditions at three time points throughout the same slide. The first time point is 240 ms into the slide, the second is halfway through (1,500 ms), and the final is 240 ms from the end of the 3-s slide. Context condition participants can be seen to fixate the car more often than the no-context participants. Figure and caption recreated with permission from Hutson et al. (2022). (Color figure online)

The biased competition model explains how task-driven goals can override visual stimulus features in determining what is attended (e.g., Hutson et al., 2017, Exp. 2B). However, a relatively unanswered question is how prior knowledge, and event models can enter this competition, and sometimes win. Imagine two different viewers of the 3-min scene illustrated in Fig. 1. The first viewer watches it from the beginning of the scene, so they know that the driving couple have a ticking *time bomb* in the *trunk of their car*. The second viewer does not start watching until approximately 1 min later in the scene, when the camera follows the walking couple as shown in sixth frame of Fig. 1, so they have no idea of either the time bomb or the driving couple. As shown in the bottom of Fig. 2, as the scene progresses, the car with the time bomb is highlighted in the event models for the first viewer. As we will discuss later in detail in the section entitled, "Stimulus features need to afford knowledge-driven or task-driven attentional control" (and Fig. 5), the car is just part of the scene background for the second viewer. SPECT assumes that competition for attentional selection will be biased differently between the two viewers, based on their different event models, and there is evidence supporting that (Hutson et al., 2017, 2022). However, SPECT also assumes that the influence of the event model is attenuated when viewers have a task-driven goal, or there is high visual saliency, competing with the event model to influence attentional selection (Hutson et al., 2017). Candidate brain areas for processing event models, which would likely enter this competition are those involved in the default mode network (DMN), a set of interconnected brain regions involved in internal thought, specifically for processing event models, such as the precuneus, the posterior cingulate cortex, and the medial prefrontal cortex (M. E. Smith et al., 2025).

### The back end

As shown in Fig. 3, the back end involves processes that involve building the *current event model* in working memory, which in turn is important for building the *stored event model* in episodic long-term memory. Understanding the back-end is important for understanding attentional selection. This is because SPECT assumes that processes affecting construction of the current event model in working memory dynamically affect attentional selection as the event unfolds. Figure 3 shows that the *back end* of SPECT has four main components: 1) the current event model in working memory; 2) episodic long-term memory, which contains the stored event model; 3) semantic long-term memory, which contains prior knowledge (e.g., schemas and scripts); and 4) executive processes, such as goal-setting, attentional control, and inhibition, which affect what information is maintained in the current event model in working memory. The back end of SPECT is informed by theories of event comprehension that were first proposed and tested in the context of reading narrative texts (Gernsbacher, 1990; Zwaan & Radvansky, 1998) and more recently further developed in the context of real-world event cognition (Kurby & Zacks, 2008; Radvansky, 2012; Radvansky & Zacks, 2011).

#### The current event model

As shown in Fig. 3, *the current event model* is assumed to be dynamically constructed in working memory. From the perspective of Baddeley’s (2000) working memory model, the current event model would use the episodic buffer. From Ericsson and Kintsch’s (1995) perspective, the current event model may use long-term working memory. SPECT also includes the critical theoretical construct of *event indices*—these are the high priority pieces of information that viewers use to construct the current event model, and are likely to be recalled later from long-term episodic memory (Zwaan & Radvansky, 1998). Event indices include *events* (e.g., actions), *entities* (people, objects), *time*, *location*, *goal*(s) (of entities), other *causal relationships* (both social and physical), and entities’ *emotions* (Gernsbacher et al., 1992). Figure 1 illustrates a number of events—for example, a time bomb being placed in the car trunk, the couple who own the car unknowingly driving off with it, and another couple walking nearby. These events typically involve hierarchically nested actions (e.g., opening the trunk, placing the bomb in it, closing the trunk), and a series of state changes (e.g., the bomb goes from outside the trunk to inside it), which unfold over time (e.g., a few seconds) and across locations (e.g., the parking lot, city streets). Monitoring these event models in working memory allows viewers to track entities, their goals, causal relationships, emotions, and the spatiotemporal framework of the unfolding narrative (Magliano et al., 2012; Zwaan & Radvansky, 1998).

SPECT draws upon the structure building framework (Gernsbacher, 1990) to describe how the current event model is constructed, and how it is later stored in episodic long-term memory as a *stored event model*. The stored event model is construed as a hierarchically organized representation of prior current event models (Zwaan & Radvansky, 1998), which are organized around the goal-structure of characters/agents (Trabasso et al., 1989). The structure building framework describes three phases of event model construction: *laying the foundation, mapping*, and *shifting*.^2^

##### **Laying the foundation**

As shown in Fig. 3, the first phase of constructing the current event model is called *laying the foundation*. The foundation of the event model contains a representation of its initial spatiotemporal context, key entities, and actions/events that are acquired within the viewer’s first few fixations (Larson, 2012). Importantly, SPECT assumes that the *gist* of a scene is critical for laying the foundation (as described above for the *front end* in terms of *broad information extraction*). A concrete example of laying the foundation in Fig. 1 is the beginning of the first event of “the villain planting the bomb in the car.” The beginning of the first event starts with a close-up of a man’s hands setting the timer on a bomb. The viewer’s first fixation would extract the information of “hands” (of a person), “holding” an inanimate entity, which may be recognized during a second fixation as a “time bomb.” The person whose hands are seen will be perceived as an “agent” (the doer of an action), who is “holding” (an action) the “patient” (the bomb). Prior research has shown that the “agent” of an action is rapidly recognized within a single eye fixation, before recognizing the “patient” (Dobel et al., 2007; Hafri et al., 2013).

Thus, all of the above information could be extracted automatically within the time frame of one to two eye fixations (roughly 300 ms each; Dobel et al., 2007; Hafri et al., 2013; Larson, 2012). The viewer’s first few fixations, which have extracted “man holding and setting a time bomb,” may activate “terrorist” or “spy” scripts from semantic long-term memory. Thus, the person holding the bomb may be given a moral interpretation (e.g., “villain”) by roughly 300–700 ms after fixating it (Ma et al., 2024).

Then, as the close-up on the villain’s hands zooms out to show more of the scene, the foundation of the viewer’s current event model will include the spatiotemporal scene gist (city, parking lot, evening; Fei-Fei et al., 2007; Greene & Oliva, 2009; Larson & Loschky, 2009; Loschky et al., 2007). The sum total of this beginning information could constitute the foundation for the viewer’s event model (e.g., a villain [agent] has set the timer [action] on a time bomb [patient], in a city parking lot [place], at night [time]). Importantly, these key pieces of information are all event indices (Radvansky & Zacks, 2014; Zwaan & Radvansky, 1998).

Laying the foundation is an iterative process. As shown in Fig. 1, in the 3-min *Touch of Evil* scene, there are at least six distinct events that require laying the foundation. Those are: 1) the villain placing the bomb in the car, 2) the first couple unwittingly driving off in the bomb-laden car, 3) the second couple walking through the city streets, 4) both couples simultaneously going through the border checkpoint, 5) the walking couple kissing. The immediately following shot (*not* shown in Fig. 1), shows a sixth event, the bomb exploding. Thus, the process of laying the foundation would occur in the first fixations at the start of each new current event model. When the viewer will lay the foundation is determined by the products of the mapping and shifting processes, described below. Finally, laying the foundation for the first event model of a narrative, which should occur during the first scene of a film, the first page of a picture story, or the first panel of a comic strip, typically produces longer processing times than subsequent ones (Foulsham et al., 2016; Gernsbacher, 1983). Similar results are well known for reading times of the first sentence in story compared with subsequent sentences (Haberlandt & Graesser, 1985). Thus, laying the first foundation represents a necessary cognitive investment that enables faster processing times later.

##### **Mapping**

Figure 3 shows a rightward arrow from laying the foundation to mapping, because the former enables the latter. Viewers develop their current event models by mapping newly extracted information onto its foundation. However, whether incoming information is mapped onto the event model’s foundation depends on how coherent and/or predictable that information is with the current event model. For example, as viewers process the events that make up the first *Touch of Evil* scene, they must judge whether the incoming subevents (e.g., villain setting a time bomb, putting the bomb in a car trunk, running away) belong together in the current event model. Two important processes support mapping.

The first process to support mapping is situational *coherence/continuity monitoring* (Gernsbacher, 1990; Radvansky, 2012; Zwaan & Radvansky, 1998). Specifically, as events unfold, people habitually assess the extent to which there is continuity in spatial locations, time, entities, causality, and the goals of agents (Huff et al., 2014; Zwaan et al., 1995). As a viewer watches the *Touch of Evil* opening scene, they will make eye movements and fixations on the villain, the car, and the owners of the car as they approach it, get in, and drive away. To the extent that the information from that series of fixations forms a coherent set of interrelated events for the viewer, the information will be mapped onto the viewers’ evolving current event model.

The second process that supports mapping is *inference generation* (Graesser et al., 1994). Bridging inferences support *backward mapping*, which often involves inferring how current events are causally related to known prior events (Trabasso et al., 1989). For example, the first shot *after* the entire 3-min 20-s long shot depicted in Fig. 1 shows the car exploding. Viewers must map the event of the car exploding to the event of the bomb being placed in its trunk, and they can do so by generating the bridging inference that the car exploded *because of* the bomb. (Viewers who have forgotten about the bomb, because it has not been visible for roughly 3 min, are surprised when the car explodes; Hutson et al., 2021).

The third process that supports mapping is *prediction generation*. The structure building framework did not emphasize *prediction* as a mapping process, but contemporary theories of event cognition assume that prediction is essential for event comprehension (Zacks et al., 2011). Prediction can be construed as *forward mapping* (using past events to predict future events). The perceived suspense of the *Touch of Evil* scene arguably requires the viewer to *predict* that the bomb will explode. The current event model in working memory is incrementally updated so long as new incoming event indices are perceived as continuously following from prior events (Kurby & Zacks, 2012) and that includes making bridging inferences to maintain coherence (M. E. Smith, Hutson, et al., 2024a, 2024b).

SPECT assumes that attentional selection is affected by the output of mapping processes in the current event model in two ways: 1) visual search and 2) predictive eye movements.

First, we consider search. When viewers experience a coherence gap during mapping in an event, they often engage in bridging inference generation to resolve it. But to bridge the gap, viewers may have to search the scene to find appropriate linking information (Hutson et al., 2018). Similarly, immediately following film cuts (i.e., transitions between separate shots), viewers make more eye movements, exploring the scene to establish event model continuity with the precut shot. Specifically, viewers make more exploratory saccades after cuts to new scenes than to cuts continuing the same scene (T. J. Smith & Martin-Portugues Santacreu, 2017). A similar pattern of eye movements during event mapping is observed in the reading literature, which shows that regressive saccades search for information needed to repair breaks in coherence during text comprehension (Calvo et al., 2001; Ehrlich & Rayner, 1983; Poynor & Morris, 2003).

Second, consider predictive eye movements. These are found in both language comprehension, and action observation (Coco et al., 2016; Eisenberg et al., 2018; Flanagan et al., 2013). For example, in the *Touch of Evil* scene, consider the second event in which the first couple gets in the car and drives away. Some viewers will predict that the couple will get into the car when they start walking towards it, and so make a predictive saccade to the car seats (e.g., Eisenberg et al., 2018).

##### **Shifting**

Figure 3 shows a rightward arrow from mapping to the third phase of constructing event models: shifting. This connection reflects that shifting occurs when mapping processes fail, prompting the viewer to shift and create a new event model. In SPECT, shifting is conceptually aligned with *event segmentation*. When ongoing information no longer maps onto the current event model, the comprehension system shifts to build a new model, and this shift is experienced as an event boundary. A great deal of behavioral and neurophysiological evidence shows that the event comprehension system parses larger events into smaller ones (Zacks, 2020). The current event model is maintained by a network of brain regions, including those that make up a subsection of the brain’s *default mode network*, known as the *midline default network core* (DN; Baldassano et al., 2017; M. E. Smith et al., 2025; Stawarczyk et al., 2021). Functional magnetic resonance imaging (fMRI) and electroencephalography (EEG) data show that neural activity patterns are stable as a viewer maps incoming information onto the current event model, but that rapid shifts in these patterns occur when shifts at event boundaries occur (Baldassano et al., 2017; Silva et al., 2019).

The *event segmentation task* is often used to identify when shifting occurs (Zacks et al., 2009). In this task, participants watch a movie, view a picture story, or read a text and indicate each time they perceive a new event has begun. We collected event segmentation data for the *Touch of Evil* scene. Figure 1 highlights the ten moments with the highest probabilities of perceiving event boundaries (depicted with red borders). Data from studies using this task indicate that event segmentation is hierarchically organized such that many fine, incremental events make up one course global event (Kurby & Zacks, 2011). Event segmentation occurs spontaneously, and viewers show high agreement in their segmentation behavior across the different levels of granularity (Zacks et al., 2010).

SPECT assumes that the processes of situational continuity monitoring, prediction, and bridging inference generation inform not only mapping, but also shifting (M. E. Smith, Hutson, et al., 2024a, 2024b). Shifting is probabilistically related to the perceived *number of changed event indices* (Magliano et al., 2001; Zwaan & Radvansky, 1998). In the *Touch of Evil* scene, shifting occurs when the villain moves away from the car, and when the man and woman get into the car. At this moment, there are changes in multiple event indices: the characters (one character leaves, then two others are introduced) and goals (one goal ends for the villain, and a new one begins for the man and woman). SPECT does not make specific claims regarding prioritization of specific situational indices in informing when shifting occurs. Shifting is also related to *prediction error*. The cognitive system is set up to support moment-to-moment predictions that events in the current event model will be spatiotemporally contiguous. When such a prediction fails, an event boundary is perceived (Kurby & Zacks, 2008; Zacks et al., 2011). A prediction error occurs when the walking couple is introduced because no content of the current event model can anticipate seeing *those particular* people. Finally, shifting is also related to a *failure of inference processes* to resolve a coherence gap (Brich et al., 2024; M. E. Smith, et al., 2024a, 2024b). M. E. Smith, et al. (2024a, 2024b) showed that at coherence gaps, there was a negative correlation between the likelihood of making a causal bridging inference to resolve the gap (based on think aloud data) and the likelihood of perceiving an event boundary (based on event segmentation data).

When a viewer *shifts* to create a new current event model, the *stored event model i*n long-term episodic memory is updated to reflect the contents of the just previous *current event model* (Radvansky, 2012; Zwaan & Radvansky, 1998). This is shown by the fact that people are less accurate at retrieving information from previous events after perceiving an event boundary (Gernsbacher, 1985; Pettijohn & Radvansky, 2016; Swallow et al., 2009). Further support comes from studies measuring fMRI and EEG brain activity at event boundaries. Specifically, shifts in neural activity patterns at event boundaries across cortex are accompanied by increases in neural activity in the *hippocampus* (Baldassano et al., 2017; Ben-Yakov & Dudai, 2011). Assumedly, boundary-evoked hippocampal activity reflects early long-term memory consolidation processes, where the contents of the previous current event model are updated in episodic long-term memory as part of the stored event model. This raises the question of whether updating an event model in episodic long-term memory only occurs at coarse event boundaries, with transitions between fine event boundaries only occurring within the current event model in working memory. One of the few studies to address this question found that EEG correlations with previously encoded stimuli were greater at coarse boundaries than at fine boundaries (Sols et al., 2017). Furthermore, memory decreased after coarse event boundaries, but *not* after each new image of an event, or page in a picture story (Gernsbacher, 1985; Swallow et al., 2009). Finally, as noted earlier, there appears to be a brief time of limited information extraction for roughly 1 s before and after an event boundary (Crundall et al., 2002; Huff et al., 2012; Ji & Papafragou, 2022; Yates et al., 2024).

### Coordination of front- and back-end processes

In Fig. 3, the coordination of front- and back-end processes is indicated by downward arrows from the back-end event model to the front-end attentional selection and information extraction processes, and by upward arrows from information extraction to the event model. Such coordination is further illuminated by considering the previously discussed front-end ambient-to-focal shift in eye movements (Pannasch et al., 2008) together with the three event model construction processes (laying the foundation, mapping, and shifting). Information extracted during the first two seconds of viewing a static scene image, during the ambient mode, likely serves to lay the foundation for the current event model. Then, the focal mode occurs from roughly 4–6 s into viewing a scene, which may correspond to the mapping process. Once the back-end shifting process occurs, and laying the foundation restarts, and the front-end ambient mode will begin again (Eisenberg & Zacks, 2016). Thus, we can give additional motivation for these front-end changes to attentional selection, from ambient mode (broad) to focal mode (narrow) in terms of the three back-end event model processes of laying the foundation, mapping, and shifting.

#### Prior knowledge

##### **Episodic long-term memory**

Episodic long-term memory is a key part of SPECT because it contains the *stored event model*. Figure 3 shows a downward arrow from episodic long-term memory to the three phases of structure building, and an upward arrow from shifting to episodic long term memory. The downward arrow reflects the fact that the contents of episodic memory can become activated in WM. This occurs to the extent that current event model information serves as a retrieval cue for the stored event model (Kintsch, 1988). Accessing the stored event model is important for laying the foundation for a new current event model because agents, entities, locations, and goals that are represented in the stored event are often reintroduced during this stage. The stored event model is also an important source of information used in mapping (Graesser et al., 1994; Kintsch, 1988) because accessing *the stored event model* supports *continuity monitoring* (Zwaan & Radvansky, 1998), and the generation of *bridging inferences* (Graesser et al., 1994) and *predictive inferences* (Loschky et al., 2015).

The Arrow going up from shifting to episodic memory reflects that the stored event model is updated when shifting occurs (Baldassano et al., 2017; Sols et al., 2017). Viewers shift to create a new event model (as indicated behaviorally by making an event segmentation response) roughly every 12 s in studies by Zacks and colleagues. Thus, when watching a feature length movie, a viewer’s *stored event model* for the entire movie may be the concatenation of a hundred or more previous current event models. Research has studied the processes involved in structuring such stored event models in episodic long-term memory, and later retrieving them (Radvansky, 2012; Radvansky & Zacks, 2014).

##### **Semantic long-term memory**

Semantic long-term memory refers to general knowledge of the structure of the world. Figure 3 shows a downward arrow from semantic memory to the three stages of structure building. Similar to episodic memory, the downward arrow reflects the fact that semantic memory can become part of the *current event model*. This happens to the extent that information in the current event serves as a retrieval cue for *semantic memory* (Kintsch, 1998). Semantic memory can be accessed during all three stages of structure building. This assumption is grounded in theories and research on both scene perception (Eckstein et al., 2006; Palmer, 1975; M. E. Smith & Loschky, 2019) and text comprehension (Kintsch, 1988, 1998; Myers & O’Brien, 1998). Both areas assume that the activation and use of semantic knowledge is necessary for recognizing and comprehending events. SPECT assumes that semantic long-term memory for types of events is represented in *schemas*, also known as *scripts* or *frames* (Barsalou, 1992; Elman, 2009; Hare et al., 2009). Event schemas represent the key information that probabilistically co-occurs in the context of events, and reflects the specific contents of event indices: *time* (e.g., of day, or of year), *location*, *entities* (people, animals, objects), and *actions/events* (both intentional and unintentional; Magliano et al., 2008). The probability that information in an event schema becomes activated depends on *constraint satisfaction* via *spreading activation* from the products of information extraction (McNamara & Magliano, 2009).

#### Executive processes

As shown in Fig. 3, SPECT includes executive processes to account for non-automatic influences on attentional selection. These executive processes include cognitive flexibility via *goal setting* (i.e., identifying and voluntarily switching between tasks), *attentional control* (i.e., volitionally paying attention to task-relevant information), and *inhibition* (i.e., intentionally ignoring irrelevant information; Diamond, 2013). Executive processes are often considered to include working memory due to its *active* and *volitional* maintenance of information (Diamond, 2013). However, within SPECT we conceptualize working memory as a separate memory process *acted upon* by executive functions, namely volitional attentional selection to encode task-relevant information into working memory.

Executive processes are thought to be important in enabling task-driven influences on attentional selection and information extraction. Task-driven influences are volitional in nature (Baluch & Itti, 2011; Theeuwes, 2018), and thus appear to be qualitatively different from experience/knowledge-driven influences, which are typically automatic (e.g., schema activation). SPECT considers executive processes (i.e., the management of goal-directed behaviors) to be part of the back end, because they operate on time scales far longer than single fixations and may occur infrequently, only when necessary due to the effort involved. Executive processes are mediated by frontal and prefrontal brain areas, specifically the dorsolateral prefrontal cortex (DLPFC) and the frontal eye fields (FEF). Both areas are involved in *attentional control*, a key executive process in SPECT. An extreme example of such attentional control is the *anti-saccade task*, in which, when a simple target appears on a screen, the viewer must make a saccade to the opposite direction (Hallett, 1978). Anti-saccades require *goal setting* by the viewer, to exert *attentional control* of their eyes, and *inhibiting* saccades to the distractor (Mitchell et al., 2002; Unsworth et al., 2004), all key executive processes in SPECT. Most people can do the anti-saccade task reasonably accurately (a sample of >2,000 healthy young men made 23% errors), but much more slowly than they make pro-saccades to the visual target (Evdokimidis et al., 2002). Importantly, making anti-saccades is more difficult when a person’s executive working memory is taxed (e.g., by doing mental math, or other working memory tasks; Mitchell et al., 2002; Roberts et al., 1994; Unsworth et al., 2004).

A more ecologically valid example of deliberate and task-driven executive processes influencing viewers’ attentional selection is visual search (Wolfe, 2021; Zelinsky et al., 2020). By definition, all cases of visual search involve the executive process of goal setting, because search is inherently goal directed. Nevertheless, in easy cases of visual search, such as the earlier example of searching for a chimney in a picture of a yard with a house (Eckstein et al., 2006), very little task-driven attentional control or inhibition are needed. However, the need for greater attentional control and inhibition increases as the difficulty of the visual search increases, because there is less guidance by prior knowledge, greater visual similarity between the target and nontargets, or greater saliency of the nontargets. The most famous ecologically valid examples are the “Where’s Waldo” images, during which the goal and target template for Waldo must be actively maintained in working memory, and saccades to similar distractor characters inhibited, to successfully locate Waldo in complex scenes (Smirl et al., 2016; T. J. Smith & Henderson, 2011). Importantly, SPECT assumes that visual search can also be motivated by the nature of the event model. This assumption is influenced by what is known about visual search in the context of reading. When readers encounter comprehension problems (which in the context of narratives, involve the event model), they engage in regressive eye movements to correct those problems (Calvo et al., 2001; Foulsham et al., 2016; Poynor & Morris, 2003; Wiley & Rayner, 2000). We assume that when visual saliency is low in the context of a dynamic event, the event model can also affect attentional selection.

Hutson et al. (2018) showed that visual search produced additional eye fixations when a coherence gap caused difficulty in the mapping process, which could be resolved based on extracting information from the pictures. This is consistent with the assumption of SPECT that event models can trigger executive processes when there is a difficulty in creating the current event model. However, an open question is whether the visual search for information to create a bridging inference in picture stories was volitional. We suspect that those fixations are not the result of effortful processing, based on prior research on text processing, which shows that many inferences needed to create a coherent event model result from passive, memory-based processes (e.g., Myers & O’Brien, 1998). Additionally, executive load when processing visual narratives that require generating bridging inferences attenuates inference processes, but does not eliminate them (Magliano et al., 2016). Clearly it is possible for comprehension problems in the event model to lead to deliberate, effortful executive control over attention to resolve them. However the visual salience of the narrative and the encoding conditions (e.g., self-paced processing) must afford that executive control (Magliano, 2024). Thus, a key hypothesis generated by SPECT, which we have tested, is that goal-driven executive attentional selection, involving visual search, occurs when there is difficulty in processing the current event model. However, such visual search will vary in the degree to which it is effortful.

Importantly, we have also shown that executive attentional control can volitionally override the impact of stimulus saliency of a dynamic film stimulus when the task is at odds with comprehending the narrative (Hutson et al., 2017; Simonson et al., 2021). In Simonson et al. (2021), participants performed one of two tasks, which were either congruent with comprehending the narrative (watching the clips in preparation to draw a four-panel comic of the narrative) or at odds with comprehending the narrative (watching the clips in preparation to draw a map of the key landmarks and their relative locations). The map task can be conceptualized as involving a high-level visual search for spatial landmarks. The results showed that participants looked more at the background of the scene when performing the map task than the comic task—thus, task-driven attentional selection overrode the tendency to look at the main characters (agents) in the center of the screen. To determine whether the map task required executive attentional resources, in another condition, participants had a secondary auditory 2-back task, while watching the silent film clips. The executively demanding 2-back dual task reduced the extent to which those in the map task looked at the background, but it did not reduce eye movements to the central part of the screen and the agents in the comic task. In sum, viewers can executively control their eye movements while watching narrative film—perhaps via selective inhibition of visually salient features around the screen center—and doing so can override all the stimulus factors in film used to guide their gaze, but it requires effortful executive control. Similar evidence of inhibitory mechanisms has been shown by Bezdek and colleagues (Bezdek & Gerrig, 2017; Bezdek et al., 2015) who showed narrowing of attentional focus and reduced activity in the default mode network during suspenseful film scenes, suggesting inhibition of irrelevant sensory features.

Conversely, during typical movie viewing, the degree to which viewers engage executive processes is unclear. The highly designed nature of most film stimuli (e.g., shot composition, framing, editing) is believed to encourage stimulus-driven (i.e., *not* executively driven) attention to the most meaningful information for understanding the movie (T. J. Smith, 2013). Nevertheless, prioritization of the bottom-up features of film scenes may actually constitute an executively maintained attentional prior. Namely, studies attempting to artificially capture gaze with sudden onsets during film viewing have failed to do so (Hinde et al., 2017). Fixation durations immediately following distractor onsets are artificially elongated, suggesting distractors are seen and processed but capture is suppressed, possibly due to their irrelevance to the film viewing task (Hinde et al., 2017). Whether such inhibition of saccades occurs volitionally via executive processes, or more automatically once an overarching viewing goal is set at the start of viewing, is unknown.

Thus, while viewers can volitionally control where they look, it unlikely their first option, because it requires cognitively “expensive” executive resources, that are slow and effortful. Instead, in everyday viewing of visual narratives, task-driven control of attentional selection may be the exception rather than the rule (Theeuwes, 2018). This reasoning leads to a key testable assumption of SPECT—namely, while viewers watch visual narratives, knowledge-driven top-down influences on their attentional selection are more common than task-driven top-down influences, which, if at odds with following the narrative, will require more executive resources and be more effortful.

## Using SPECT to study the role of event models in attentional selection in scenes

SPECT provides a theoretical framework for generating testable hypotheses about how moment-by-moment real-world scene understanding, formalized in terms of event model processes, guides attentional selection. SPECT assumes that the extent to which event models exert a knowledge-driven top-down influence on attentional selection depends on their competition with bottom-up features (visual salience, or medium-specific features). Specifically, top-down effects of the event model should be stronger when bottom-up visual features are weaker. We next review studies demonstrating this push-and-pull between the stimulus and the viewer’s current event model to control of attentional selection. We show this during both real-world events, and visual narratives, including film clips, slide shows, and picture stories, which vary in their bottom-up features. Sequential visual narratives (picture stories or slide shows of films) are static, whereas films are dynamic. The dynamic nature of film introduces *motion*, a potent *stimulus feature* that strongly competes for attentional selection. Accordingly, SPECT predicts that top-down influences of the event model on attentional selection will be more likely in *sequential* visual narratives (e.g., picture stories, or slideshows) than in filmed narratives. Also important to note in these studies, the need for volitional tasks beyond basic comprehension was minimal.

### Stimulus features must afford knowledge-driven or task-driven attentional control

Films, by definition, move. In doing so, they exert the maximum potential to guide viewer attention via visual saliency (Carmi & Itti, 2006; Mital et al., 2010). Furthermore, filmmakers frequently make cuts between shots (i.e., periods of continuous recording of a single camera), immediately after which viewers look at the center of the screen (T. J.Smith, 2012a; Wang et al., 2012). Together, these two stimulus factors (the saliency-engendering feature of motion, and the medium-specific feature of cuts) minimize the viewer’s opportunities to use their event models to guide their attention. Studies of gaze behavior during film viewing are consistent with SPECT’s assumption that bottom-up features in filmed narratives (e.g., motion) decrease the likelihood that event models will exert a top-down influence on attentional selection (Hutson et al., 2017; Loschky et al., 2015). Loschky and colleagues (2015) manipulated the viewing context of participants who watched a short film clip from a James Bond film, by showing the preceding 3 min of the clip, in the context condition, or not, in the no-context condition. The manipulation created a strong difference in viewers’ event models for the clip, as measured by viewers’ think-aloud protocols, event segmentation, and predictions of what would happen next. But these big differences in event models produced only small (though statistically reliable) differences in eye movements. The only moment when there was a significant effect of viewers’ event models on their attentional selection was in a shot with virtually no motion. The authors concluded that this strong attenuation of the effect of the event model on attentional selection, which they called *the tyranny of film*, was due to the nature of the film stimulus. Namely, the clip used Hollywood style filmmaking techniques to direct viewers’ attention, including editing with many short (2 s) shots, showing only a single character, mostly at the center of the screen. This left little room for viewers’ event models to influence their attentional selection. Furthermore, there was no key *target of attention* to differentiate the attentional selection of viewers having different event models.

Thus, a follow-up study by Hutson et al. (2017), showed participants the opening scene of Orson Welles’ film *Touch of Evil* (Welles & Zugsmith, 1958), which we have discussed at length above. A key medium-specific feature of this scene is that it consists of a single, continuous 3-min shot with no cuts. Thus, it was predicted that this movie clip would provide greater opportunity for viewers’ event models to influence their attentional selection. Experiment 1 manipulated viewers’ event models through context. Viewers in the context condition watched the entire clip, while those in the no-context condition started watching after the bomb was hidden in the car trunk (i.e., they did not know about it). As one would expect, participants in the context condition were much more likely to predict at the end of the scene that the bomb would explode—showing they had a very different event model for the scene than participants in the no-context condition (who predicted, say, that the two couples would have dinner together). However, there were no significant differences in any of the eye movement variables tested between the two conditions—fixation durations, saccade lengths, or fixations of the car. These results again supported the tyranny of film hypothesis, that bottom-up features of the film scene guided viewers’ attention despite large differences in their event models.

Hutson et al. (2017) hypothesized that the *No-context *participants may have continued looking at the car (the *target of attention*) because they considered the driving couple as *protagonists*,^3^ or more formally, *agents* (Zwaan & Radvansky, 1998). Experiment 2 tested that hypothesis by having the no-context condition participants start watching the film about half way through the clip, when the *walking couple* were at the center of the screen, thus establishing them as the *agents* for those participants. Several seconds later, the walking couple passed by the car, which was parked near an intersection (as shown in Fig. 2). Here, it was hypothesized that the no-context participants, who had never seen the couple in the car before, would not consider them as agents, but instead part of the scene background. Consistent with that hypothesis, for the first 8 s that the car was on-screen, participants in the *new no-context condition* were significantly less likely to fixate the car than the context participants. Hutson et al. (2017) called this the *agent effect*. Namely, entities treated as protagonists (i.e., agents) had higher priority in their current event model, which influenced their attentional selection. Nevertheless, after those first 8 s, the couple in the car became part of the ongoing action, and thus were treated as agents by participants in both conditions, like the walking couple. Thus, the difference in attentional selection between the context and no-context conditions due to the agent effect was very short lived.

To test whether the broader lack of top-down guidance was limited to knowledge-driven top-down processes, or also extended to task-driven top-down processes, in Experiment 2B, Hutson et al. (2017) added a condition in which viewers were given the task of watching the film clip in preparation to draw a detailed map of all the landmarks and their spatial relationships afterwards. Note that one could predict that this task would guide viewers’ attention to the *background* of the scene, rather than the main characters, similarly to Lahnakoski et al.’s (2014) “interior decorator” perspective discussed earlier. Indeed, the authors found a clear effect of top-down task-driven guidance of viewers’ attention to the film’s background elements. Importantly, the map task was at odds with understanding the narrative—namely, the map task required viewers to ignore the main characters at the center of the screen, and instead study the background. Consistent with this claim, participants in the map task condition, who saw the bomb placed in the car, were far less likely to make a prediction about the bomb exploding than those who simply watched for comprehension (i.e., 13% vs. 50%). This strongly suggests that the map task interfered with comprehending the narrative. A follow-up study (Simonson et al., 2020) replicated and extended this result to a set of eight film clips, and two viewing tasks: watching the clip in preparation to do 1) the map task versus 2) a comic drawing task (i.e., watch the clip in preparation to convey the film clip narrative in a four-panel comic). Attentional selection differed between the tasks, with only the map task producing fixation patterns away from the main characters (entities).

The above results raised the question: Why were the effects of the event model on viewers’ attentional selection relatively small and short lived (Hutson et al., 2017; Loschky et al., 2015)? As discussed earlier, one of the strongest bottom-up predictors of attentional selection in film is motion (Carmi & Itti, 2006; Mital et al., 2010). This led to the prediction that motion may wash out effects of the viewer’s event model on attentional selection while watching film. To test this motion hypothesis, Hutson et al. (2022) used the same *Touch of Evil* scene, but eliminated motion by transforming the film clip into a slideshow (i.e., one frame sampled from the film every 3 s was presented for 3 s to maintain the rough timing of events). All other aspects of the experiment and conditions were the same as Hutson et al. (2017, Exp. 1). When they removed motion from the visual narrative, they found the predicted effects of different event models on viewers’ attentional selection (Hutson et al., 2022; see also Pedziwiatr et al., 2023). For example, as shown in Fig. 5, viewers in the context condition were more likely to fixate the car with the bomb, and this difference in attentional selection was fast, occurring *from viewers’ first saccade on an image showing the car*. This speed was likely due to the fact that participants already had an event model that prominently included the bomb in the car, which was built earlier during the context manipulation.

**Fig. 6:**
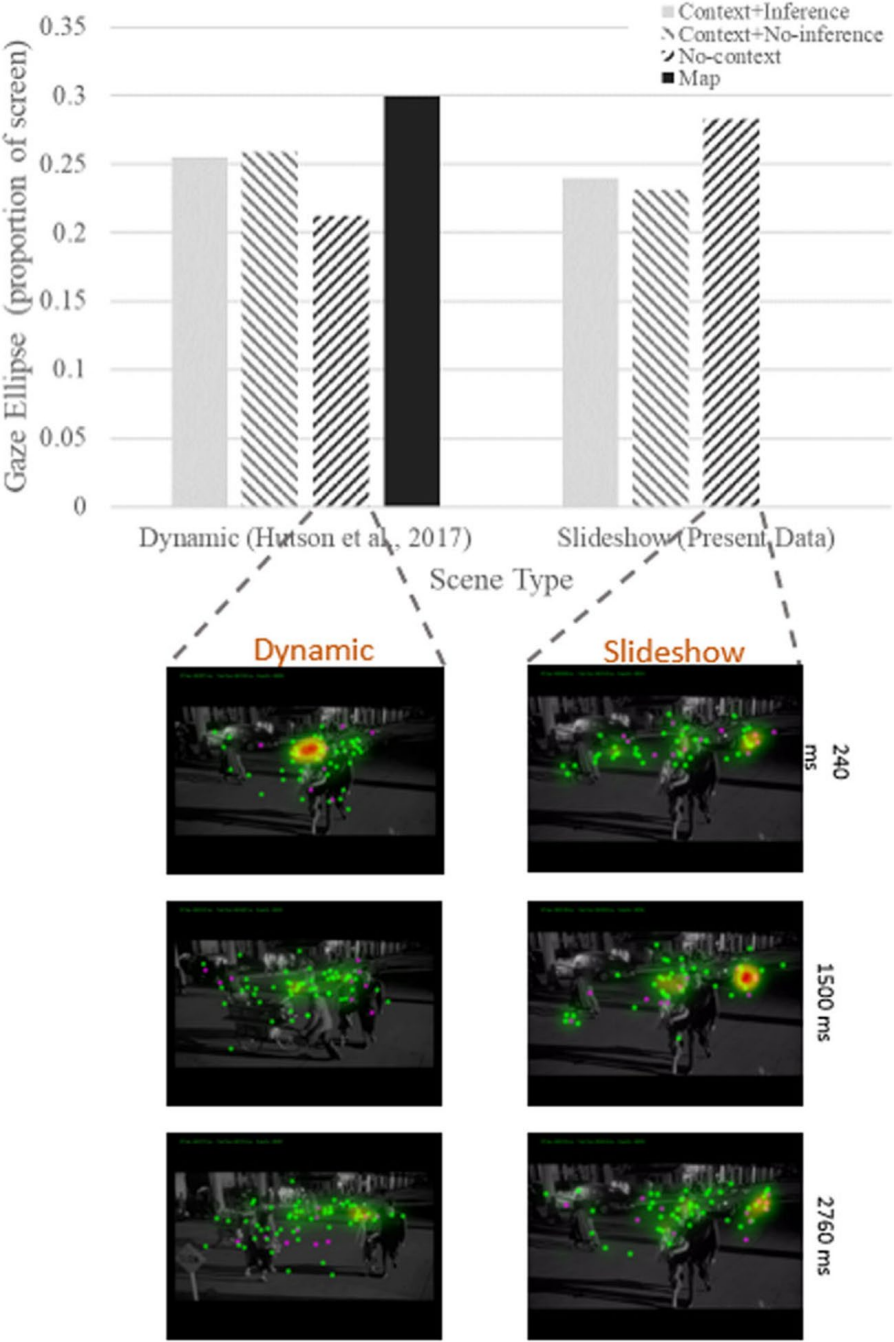
Above: Gaze ellipse areas for the Hutson et al. (2017; dynamic [film]) versus the Hutson et al. (2022; slideshow). The ellipse area is the proportion of the screen the ellipse covers. Context + inference is solid light gray; context + no inference is a diagonal pattern slanted downwards from left to right; no-context is diagonal pattern slanted upwards from left to right; map task is solid black. Below: The heatmap images illustrate the gaze differences between the No-context conditions of the dynamic (left) and slideshow (right) presentations. For the slideshow, the image is from 240 ms into the 3-s slide. For the dynamic, the first image is the same frame as the one in the slideshow. The heatmaps show that no-context viewers’ gaze in the slideshow condition was more dispersed than in the dynamic (movie) condition. (Figure and caption recreated with permission from Hutson et al., 2022.) (Color figure online)

Furthermore, when comparing viewer eye movements in the dynamic (film) and slideshow experiments, it was found that in the context condition viewers’ gaze covered a similar proportion of the screen, but for the no-context condition there was a difference (Fig. 6). Specifically, participants in the no-context condition in the slideshow experiment had less clustered gaze (i.e., their fixations covered a larger proportion of the screen) than participants in the film experiment. Thus, the attentional selection of viewers with a *weak* event model was more influenced by *bottom-up stimulus motion.*

It is also important to consider how the *temporal processing constraints* of event models may influence attentional control. Attention has to be optimally deployed by viewers to identify and encode the most important information at any particular moment. This is constrained by the pace of information flow, the viewer’s speed of information extraction, and temporal constraints on overt attentional selection via eye movements. During dynamic scenes, the impetus is likely to be placed on the scene dynamics driving this process due to the inability for viewers to stop the flow of information. If viewers are given the opportunity to control the temporal flow of information in a dynamic scene, for instance by using a self-paced slideshow presentation, they have been shown to slow the presentation of frames at event boundaries (Hard et al., 2011). They have similarly been shown to slow the presentation during moments critical to spontaneous theory of mind reasoning, which would constitute a recursive event model—namely, the viewer’s event model of a character’s event model (Cabañas et al., 2026). For example, when watching silent film scenes in which a character is ignorant of an important piece of narrative information that viewers know, such as the fact that the character’s fiancé is embracing her brother rather than a secret lover (known as *dramatic irony scenes*), the discrepancy between the viewer’s knowledge and the viewer’s representation of the characters’ knowledge leads to a slowing down of self-paced film viewing at critical dramatic moments (Cabañas et al., 2026). The difference in the viewer’s event models does not change processing overall, just at the moments when the recursive event model is relevant to the current scene. This context effect is further emphasized by changes in gaze behavior during these critical frames. Dwell time on characters and objects critical to the viewer’s event model of the scenes’ dramatic irony are longer for viewers who perceive the dramatic irony (the recursive event model). This evidence directly supports SPECT’s predictions that the current event model can directly influence front-end processing but only if the stimulus affords such control (as the self-paced silent film sequences do), when such control is relevant to the event model.

Taken together, these results testing hypotheses generated by SPECT indicate that the top-down influence of the viewer’s event model does guide attentional selection in scenes, but only when bottom-up saliency (e.g., from motion signal) is weak. For the top-down factor of task, it must also be strong and at odds with following the narrative to manifest in differential attentional selection in film. Otherwise, attention to information important to comprehending the narrative will not differ between top-down driven and stimulus-driven elements.

### Studies showing that event model processes of mapping and shifting affect attentional Selection

In this section we discuss studies that show that the back-end sub processes of mapping (here, bridging and predictive inferences) and shifting (event segmentation) affect attentional selection. One common feature of these studies is that the impact of salient features on attentional selection is relatively lower than in studies involving commercially produced movies described above. These studies either used picture stories or videos of actors performing everyday activities as stimuli.

#### Bridging inferences

Hutson et al. (2018) investigated the role of viewer’s event models on attentional selection in visual narratives. To do that, they tested how the *mapping process* of *bridging inference generation* influenced attentional selection in wordless sequential picture stories (e.g., Mayer, 1975). They identified picture sequences that showed a sequence of causally related events that consisted of a beginning state (Boy starts to run down a hill), a bridging event (Boy trips on a tree root), and end state (Boy falls in a pond). They created coherence gaps by manipulating the presence/absence of the bridging event, which when absent would require inferring that event (e.g., the boy fell because he tripped on something). They verified that participants could infer the missing bridging events using a think aloud task. They then collected eye movement data. Consistent with prior research that shows that the computational effort needed to compute inferences requires time (e.g., Magliano et al., 2016), they found longer viewing times on end-state pictures when the bridging events were absent than when they were present. Importantly, however, they found that this occurred because viewers made approximately two more fixations (11 vs. nine fixations) on the end-state pictures when the bridging-events were absent than when they were present. Moreover, they found that these extra fixations were more likely to go to regions of the end-state pictures that were empirically determined to be more informative for generating the bridging inferences than regions that were less informative. They interpreted these findings as suggesting that the perceived need to generate a bridging inference during the mapping process influences attentional selection processes. Specifically, viewers search for content in the pictures to support the needed bridging inference. Thus, mapping processes in the current event model influence attentional selection.

#### Predictive eye movements

As mentioned previously, viewers make predictive saccades to objects that they infer actors will interact with before the actor does so (see Gredebäck & Falck-Ytter, 2015, for a review). Wahlheim et al. (2022) tested this hypothesis by showing participants videos of an actor performing everyday activities on two fictional days. Some of the actions repeated on the second day, while others changed. For example, on the first day viewing, the actor may have opened the closet to retrieve a *bath towel*, but later, on the second day, the actor opened the same closet to retrieve a *hand towel*. Replicating prior work (Eisenberg et al., 2018; Flanagan et al., 2013), Wahlheim et al. (2022) found that viewers made a predictive eye movement to objects before they were touched by the actor on both days. More importantly, the viewers who recalled the action from the first day made a predictive eye movement to the predicted Day 1 object on the second day. This would be based on their stored event model in episodic long-term memory for actions from the first day. As shown in Fig. 3, this is consistent with the bidirectional arrows between the stored event model in episodic long-term memory and the current event model in working memory. Thus, viewers who retrieved information from their stored event model (from Day 1) on the second day, generated predictions based on their stored event model, which influenced their attentional selection.

#### Event segmentation

There is also evidence that event segmentation affects attentional selection. Specifically, the likelihood of making a predictive eye movement decreases at event boundaries (Eisenberg et al., 2018), consistent with the idea that viewers shift when the next action becomes less predictable. Instead, viewers fixate more on actors’ hands at fine event boundaries, to see what they will do (i.e., between stages within a larger goal such as reaching for a piece of clothing while doing laundry). Conversely, viewers fixate on actors’ faces more at coarse boundaries (i.e., signifying the change from one goal to another, such as finishing doing laundry and sitting down to read). The latter suggests that viewers are trying to infer the actor’s next goal from their facial expressions or gaze direction (T. J. Smith, 2012b). Preliminary research also suggests that event boundaries affect pupil dilation, gaze similarity, and the shift in attentional selection from the ambient to the focal mode. For instance, M. E. Smith, et al. (2024a, 2024b) found that pupil dilation and gaze similarity increases at event boundaries. This is in part because viewers shift into the ambient mode of processing at event boundaries, as they lay the foundation of the new current event model (Eisenberg & Zacks, 2016), like how viewers enter the ambient mode at the onset of a new picture (Pannasch et al., 2008). Viewers then return to the focal mode of processing within events, as it becomes easier to map incoming information onto the current event model (Eisenberg & Zacks, 2016). Together, these results are consistent with hypotheses generated from SPECT that failures in both 1) predictive processing and 2) coherently mapping incoming information onto the current event model cause viewers to shift and lay the foundation of a new current event model. At that moment, viewers cycle back to the ambient mode of processing, to lay the foundation for the next event model. However, research on this topic is quite limited, and many questions remain about how the active construction and maintenance of an event model, such as that proposed within SPECT, may influence attentional selection at event boundaries. A key question is the degree to which this shift involves task-driven control, or relinquishing control to the visual salience of the scene.

### Developing a systematic approach to studying the effects of event models on attentional selection in film viewing

Most of the research testing SPECT has been in the context of narrative films, which, by definition, are created by highly skilled filmmakers to prioritize visual salience in attention guidance over other factors, such as the viewer’s current event model, which might lead to idiosyncratic viewing. In the context of these studies, we have found robust evidence for the tyranny of film in narrative movies (i.e., large differences in viewers’ event models, but small differences in their attentional selection), suggesting that visual salience has a greater priority than event models on attentional selection in film viewing. This is because in conventional film clips, there is a strong congruence between the high visual saliency of the target and its importance based on the event model. This makes attentional selection of the target automatic and effortless, which allows the viewer to easily continue following the story (T. J.Smith, 2012a; T. J. Smith et al., 2012). In such cases, the target may reasonably be said to “pop-out” for the film viewer, similarly to high saliency targets in visual search, which are found effortlessly (Braun & Julesz, 1998; Wolfe & Horowitz, 2004).

Thus, an important methodological development for testing hypotheses from SPECT has been to develop a systematic approach to investigating the effects of the event model on attentional selection in film clips. Therefore, to isolate the top-down effect of the viewer’s event model on their attentional selection, we must find example film clips, or make them, which have *a dissociation between the event model and bottom-up saliency.* Taking this idea, we have developed two criteria for situations in which pre-existing film clips can be used to show the impact of the viewer’s event model on their attentional selection while watching them (Chandran et al., 2022, 2024):

#### Criterion 1

The target of attention should be prioritized by a rich event model. The differences in event models that guide attention to the target need to be measurably distinct in terms of event indices, producing *differential elements—*namely, different event indices (Zwaan & Radvansky, 1998) and causal criteria (van den Broek, 1990) between two distinct event models created in the *no-context* versus *context* conditions. Participants in the no-context condition view only what we call the *common viewing period* clip. Conversely, participants in the context condition first view a context manipulation clip before watching the common viewing period clip. The differential elements can be identified using a content analysis of viewers’ written predictions of what will happen next at the end of the common viewing period clip. When multiple differential elements emphasize the importance of the target of attention, we predict that the viewer’s event model can more strongly influence their attentional selection of that target.

To further test Criterion 1, we can ask the participants to show us what is the most important thing at each moment in the common viewing period clip, by having them hover their mouse over it while watching the clip. These mouse positions over time can then be used to generate an aggregated dynamic *importance map*, based on viewers’ event models, for each moment in the common viewing period clip. The importance map is broadly similar to a meaning map (Hayes & Henderson, 2022a; Henderson & Hayes, 2017), but importantly differs in that it reflects the judged importance of scene regions *derived from the viewers’ event models*, for a dynamically changing narrative or real-world event.

Criterion 1 may also be accompanied by necessary subconditions for meeting it:

- There is a *minimum number* of differential elements, or a *minimum subset of critical differential elements*, needed for the viewer’s event model to significantly influence their attentional selection.
- There is a *minimum threshold of time* required for *laying the foundation* of the event model and for the *key differential elements* in the viewer’s event model to significantly influence their attentional selection.

These subconditions also need to be tested.

#### Criterion 2

**Fig. 7:**
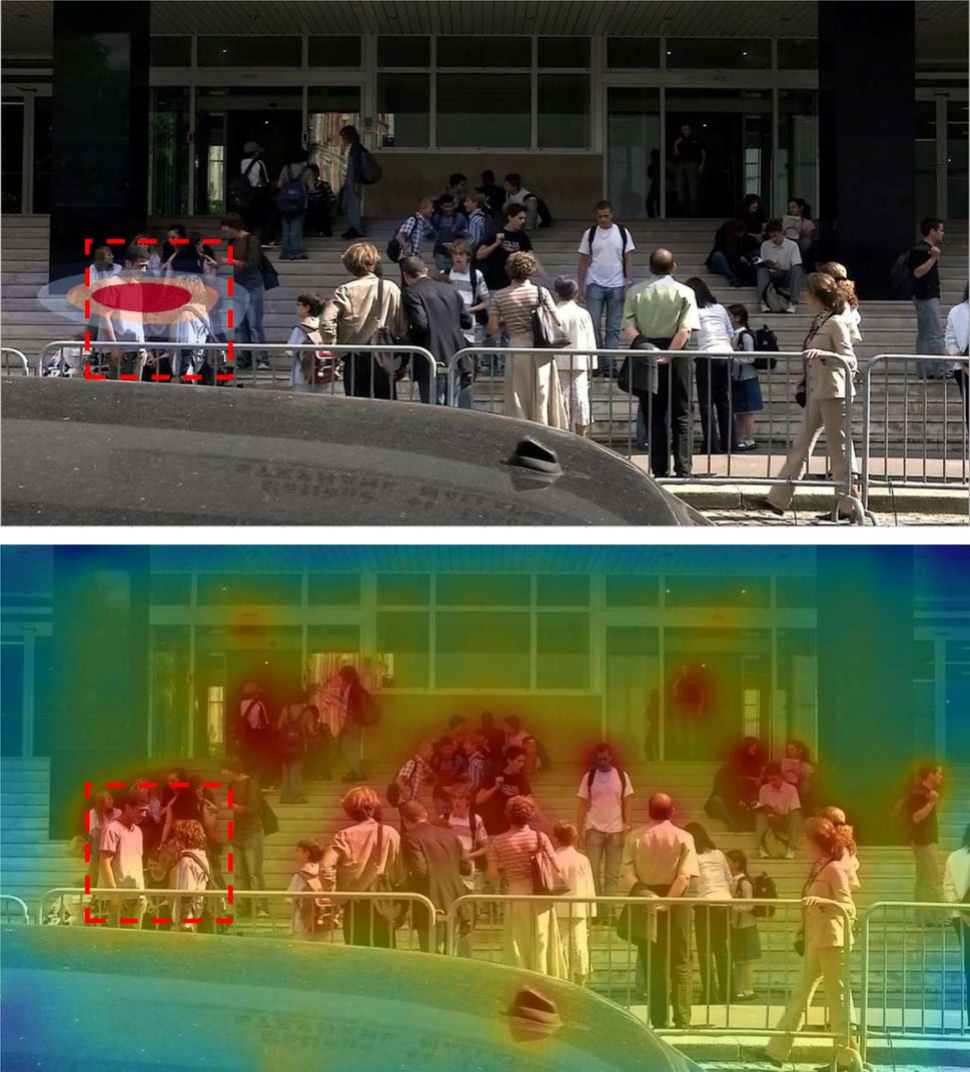
Above: Importance map for a frame from the closing shot of *Cache* (Berger et al., 2006). Warmer colors correspond to a greater proportion of participants who hovered their mouse over that region. Below: Saliency of the different locations, with warmer colors corresponding to higher visual salience in the frame from *Cache,* as estimated by the DeepGaze IIE saliency model (Linardos et al., 2021). Note that the target of attention (shown in the red area of interest), which should be deemed important in the target event model, is less salient than many other regions in the scene, particularly the cluster of boys near the exact center of the screen, etc.

The target of attention should have *relatively low visual saliency *(as measured using a state-of-the-art model), so it will not reflexively pull the viewer’s attention to the target, despite having a strong event model. The following are some, but not all, of the conditions that can reduce the saliency of the target of attention in a film clip:

- The target is not moving, nor does it suddenly appear, since motion and sudden onsets have high visual salience (Carmi & Itti, 2006; Mital et al., 2010; Theeuwes et al., 1999). If the target is moving, other surrounding things should also be moving, so the target is similar to everything else (e.g., as shown in Fig. 7).
- The target is not in the center of the screen, since the center bias (Dorr et al., 2010; Kummerer et al., 2017; Tatler, 2007) means that viewers will most frequently attend there (e.g., Fig. 7).
- The target is not a person or other animate entity, since people and animals capture attention (Fletcher-Watson et al., 2008; Humphrey & Underwood, 2010; e.g., the car trunk in Fig. 2). If the target is a person, there should be many people (e.g., a crowd), so the target is in competition for attention with the other people in the scene (e.g., Fig. 7).
- The target is not in camera focus, since blurred items are less salient (Enns & MacDonald, 2012; J. J. Peterson, 2018), but if it *is* in focus, *many other things should also be in focus,* so it is similar to everything else.

In sum, the saliency of a target of attention should be no greater than other regions of the scene in a saliency map (e.g., Fig. 7, bottom). Not all four of the above listed subconditions (a–d) need to be satisfied to achieve this second criterion, as long as it satisfies enough of them. At this point, we do not know the relative saliency weights of each of the above four sub-conditions, though we have listed them in a predicted order of precedence for attentional selection based on our above review.

When both criteria are satisfied, the target of attention is important for the viewer’s current and stored event models, but it is not visually salient enough to automatically attract the viewer’s attention. This will create a dissociation between a behaviorally measured importance map and a computationally measured salience map. The closing shot of the film *Cache* (Berger et al., 2006) meets both criteria as shown in Fig. 7 and is one film clip out of 10 used in a larger study (Chandran et al., 2024). The target of attention in Fig. 7 is indicated by a red rectangular area of interest in both the top and bottom images of the figure.

To evaluate whether the *Cache* clip meets the first criterion of having *quantifiably distinct event models* between the context and no-context conditions, we used the abovementioned context manipulation. For this clip, there are two targets of attention in the area of interest, two boys. The context condition introduced the two boys as characters (*differential entities*: an older teenage boy and a younger one). The context manipulation further indicated that there was a serious conflict between the boys’ fathers, leading the older teenage boy to seek revenge on the younger boy’s father (*differential goals*). There were several other differential elements as well (Gernsbacher et al., 1992; van den Broek, 1990; Zwaan et al., 1995).^4^ We then analyzed participants’ predictions of what would happen next in terms of whether they included the differential elements, and found substantial differences in the expected direction (Chandran et al., 2022). To provide converging evidence of the differential elements, translated to image locations over time, we used the abovementioned importance judgment task using mouse hovering (Chandran et al., 2024). This mouse data were used to create a dynamic importance map over the entire common viewing period, for both the context and no-context conditions. The top of Fig. 7 is an illustrative moment in the shot used in the study. It shows the importance map from the context condition for 30 participants. This clearly shows that for that moment in the clip, the targets of attention in the area of interest were judged the most important things in the scene, and this moment illustrates the general trend for the clip. In the no-context condition, no participants hovered their mouse over the dynamic area of interest illustrated in Fig. 7, but instead distributed their importance judgments across the image, with a strong bias to the center of the screen. All the other nine out of the 10 clips similarly showed substantial differences between context and no-context conditions (Chandran et al., 2024).

The bottom half of Fig. 7 shows a state-of-the-art saliency map of the same moment in the shot (Linardos et al., 2021). The two boys can be seen to have no greater saliency than the many other people in the image. Thus, this meets the second criterion for showing an effect of the viewer’s event model on their attentional selection in this film clip.

Follow-up experiments using the 10 film clips have measured viewer’s eye movements in the context versus no-context conditions (Chandran, 2026). This is a definitive existence proof test for whether different event models can produce different attentional selection while viewing commercially produced film clips. Having found such differences in attentional selection based on the context manipulation, it more clearly defines the conditions needed to overcome what we have called the tyranny of film.

Note, however, that the importance judgment task used to make the importance maps is metacognitive in nature—it asks participants to communicate a conscious judgment with their mouse. Such judgments are task driven and likely involve executive processes, such as inhibiting hovering over things deemed irrelevant to one’s stored event model. Conversely, we did not expect viewers’ eye movements to be so cleanly differentiated by the context manipulation, as viewers’ only task was to comprehend the film clip in preparation to make a prediction of what will happen next. Furthermore, in the no-context condition, unlike mouse hovering in the importance judgment task, viewers’ eye movements were more likely to be influenced by random fluctuations in their impoverished event model, and thus by random chance they might fixate on the targets of attention as they search for meaning. Nevertheless, the video clip from *Cache* presents a strong case of meeting the two criteria for showing the effect of viewers’ event models on their attentional selection while watching film. We preregistered a study to test the hypothesis that viewers’ eye movements differ as a function of their event models when both criteria are met (Chandran et al., 2026), and we have found strong support for it (Chandran, 2026).

## Discussion

In this paper we have discussed refinements to SPECT regarding how the current event model influences attentional selection. In this discussion, we specify the factors that influence attentional selection, underlying mechanisms by which the event model influences attentional selection, and discuss some future directions for the development and testing of SPECT.

### The competition between visual saliency versus task- and knowledge-driven top-down processes for attentional selection in scenes

Our review of the relevant literature has pointed to a hierarchy of strength of influence on attentional selection in dynamic scenes and visual narratives. There are several factors that interact in determining this hierarchy. Those factors include 1) top-down and bottom-up influences, and the two subtypes of top-down influences (task-driven vs. knowledge-driven); 2) the type of stimuli (i.e., still images, vs. dynamic images, vs. film). In most cases, these influential factors are not in conflict with each other and hence could be contributing equally to attentional selection. For example, if a viewer were given a task to watch and understand a well-directed Hollywood-style film, the stimulus features are designed to drive attention to the same event model-driven area in the scene, which also matches their current goals of the task.

Overall, task-driven top-down influences seem to be at the top of the hierarchy; whether in still images (Einhauser et al., 2008; Henderson et al., 2007; Underwood & Foulsham, 2006) or in filmed events, if their composition provides the time and space for scene exploration (Hutson et al., 2017; Simonson et al., 2021; T. J. Smith & Mital, 2013). Dynamic scenes that are composed to focus attention on key features and limit individual exploration, such as movies or video clips, have been shown to allow less expression of the task influences (Taya et al., 2012) and the event model (Hutson et al., 2017; Loschky et al., 2015). Visual saliency seems to be next in the hierarchy, being more influential than knowledge-driven top-down processes, particularly in filmed events (Hutson et al., 2017; Loschky et al., 2015), and with still images in some cases (Kaakinen et al., 2011; Underwood & Foulsham, 2006), but not in others (Hutson et al., 2022). Knowledge-driven top-down influences, such as schemas or the viewer’s event model, seem to be last in the hierarchy, but can win the competition for attentional selection if 1) the visual saliency is low enough, such as in still images (Hutson et al., 2018, 2022) or 2) for brief moments in dynamic images when motion is essentially absent (Hutson et al., 2017, Exp. 2 A, 2022; Loschky et al., 2015), or 3) if the event model is specifically relevant (Cabañas et al., 2026). As such, the literature review clearly indicates that the extent to which factors lower in the hierarchy can affect attentional selection is contingent upon how salient visual features vary across and within visual media.

There are various ways one can talk about the hierarchy. It can be the proportion of variance in fixation locations that are explained by each factor, which is the basis of our comparisons above. It can also be the time course of attentional selection. Saliency and knowledge-driven factors, including the event model, are fast, while task-driven executive control is slow (for a review, see Theeuwes, 2018). Thus, in the absence of a task, both saliency (Carmi & Itti, 2006; Mital et al., 2010) and knowledge-driven selection (Hutson et al., 2022; T. J. Smith & Martin-Portugues Santacreu, 2017; Valuch et al., 2017) can rapidly influence the first saccade in a scene, with semantics having more of an influence after roughly 2,000 ms (Carmi & Itti, 2006). But in the long run, task-driven attentional selection tends to overcome the influences of saliency (Einhauser et al., 2008; Henderson et al., 2007; Hutson et al., 2017; Simonson et al., 2021; T. J. Smith & Mital, 2013; Underwood & Foulsham, 2006).

While our review points to ranking the influences that impact attentional selection in this way, future research is needed to quantify the relative strengths of influence of each of these factors. The studies that tested SPECT and informed this proposed ranking thus far have characterized the influences of the factors affecting attentional selection hierarchy in binary terms (i.e., a rich event model, or motion, are present vs. absent). The work we have done with visual narratives has until recently not used saliency models to measure visual saliency in order to quantify its strength, relative to that of the knowledge-driven top-down influence of event models. Instead, those studies have simply held the visual stimulus constant while varying the strength of the event model, and determined whether the event model produced a significant effect on viewers’ gaze. Thus, future studies of the effects of the knowledge-driven influence of event models on viewers’ gaze, versus that of visual saliency, need to quantify both the strength of the event model (e.g., varied in terms of event indices, and measured in terms of importance judgments) and visual saliency (i.e., measured using saliency models) in order to quantitatively compare the relative strengths of each (e.g., see Chandran, 2026).

### Mechanisms for the current event model to influence attentional selection in scenes

In this article, we introduce three new mechanisms within SPECT by which the current event model can influence attentional selection: 1) the ambient-to-focal shift as event models transition from laying the foundation to mapping incoming information; 2) the working memory contents of the current event model during both laying the foundation and mapping; and 3) visual search-like attention deployments during mapping or shifting to create a new event model. Below, we discuss key topics that need to be investigated to further develop and test these mechanisms in SPECT.

A first mechanism through which the event model influences attentional selection is via the routine of the ambient-to-focal shift of eye movements during laying the foundation and mapping for the current event model (Eisenberg & Zacks, 2016; Pannasch, 2014; T. J. Smith, 2012b). This routine seems to be triggered by shifting to create a new current event model (Eisenberg & Zacks, 2016; Pannasch, 2014; T. J. Smith, 2012b). Shifting to the ambient mode of processing at event boundaries may facilitate laying the foundation because key global information is quickly extracted at the start of a new event. Reverting to the focal model of processing within events may facilitate mapping detailed information onto the event model as it is extracted. An important question, however, is the degree to which the ambient-to-focal shift is uniquely tailored to each new event. Is each saccade sent to the ideal location for extracting the information needed (Najemnik & Geisler, 2005) to fill out the new current event model? If so, this would implicate the event model’s influence from its nascent beginning, perhaps based on the stored event model, and associated schemas or scripts. Alternatively, is the ambient-to-focal shift more of a standard routine that can be followed automatically, much like demonstrations of automated eye movements in the reading literature (e.g., “Z-reading”; Vitu et al., 1995)? Such reading-like eye movements have also been successfully simulated by a computational model of eye movements based on the architecture of the midbrain structure, the superior colliculus (Vitu et al., 2021). Furthermore, the same model, when provided with a priority map indicating a task-driven goal, also successfully simulated eye movements during categorical visual search (e.g., search for a “clock” in a picture; Adeli & Zelinsky, 2018). Further research could tease apart these two possibilities by assessing the degree to which the ambient-to-focal shift is differentially affected by the specifics of the viewer’s current and stored event models (e.g., their goal structure).

A second mechanism through which the current event model can influence attentional selection is through the fact that it is created, processed, and stored in working memory. We previously mentioned the well-attested finding that the contents of working memory (e.g., objects or object features) are privileged for visual attention (Bahle et al., 2018; Olivers et al., 2006; Soto et al., 2005). Critically, the contents of working memory are maintained in a heightened state of activation (Cowan, 1993), and event indices in the event model in working memory are highly prioritized in comprehension and memory (Radvansky & Zacks, 2014; Zwaan & Radvansky, 1998). The biased competition model proposes that working memory retention requires that the perceptual representation of the remembered information be maintained in a sustained level of activation. Subsequent processing that shares the preactivated features is facilitated, and this primes spatial selection toward features that are similar to the information held in working memory. Such a mechanism is consistent with the previously discussed agent effect from Hutson et al. (2017). Namely, viewers are more likely to fixate entities that have been introduced earlier in an event (i.e., agents) than entities that have not been previously introduced. This is because viewers have a representation of the agents as part of the ongoing event model in working memory. The agent effect is one example of how situational indices of event models may affect attentional selection. This raises a key unanswered question: Is this influence of event indices on attentional selection largely passive in nature, or does it require effort? There has been a related debate concerning event models for narrative texts as to the relative roles of passive, memory-based processes (e.g., spreading activation) and effortful, constructive processes (e.g., explanation; Graesser et al., 1994; Long & Lea, 2005; Myers & O’Brien, 1998). The contemporary perspective is that much of the heavy lifting in constructing event models in working memory involves passive mechanisms (Kintsch, 1998), but that effortful constructive mechanisms are important when there are difficulties in comprehension (Chi et al., 1989). Further targeted research on this topic needs to be conducted.

A third mechanism by which the event model influences attentional selection is by engaging in visual search during mapping and shifting to create a new event model (Hutson et al., 2018). This should typically occur when repair of the event model is needed—specifically, difficulty in mapping incoming information to the current event model. This comes from the aforementioned evidence that viewers search for inferentially relevant information during the mapping process, when they need to generate a bridging inference to maintain coherence of the in-coming information with their current event model. This would be a case of an effortful constructive process as described above. Note also that such a search implies goal setting, an executive process, but how often such search rises to the level of conscious awareness is an open question. We speculate that any time the viewer’s current event model requires interrogation of a scene for specific sorts of information, either when needing to lay the foundation for a new event model, or when mapping information to the current event model, categorical visual search processes may be engaged (Zelinsky et al., 2020). Such searches may even produce anticipatory/predictive saccades when the viewer’s event model leads to a prediction of what an entity will do next (Eisenberg et al., 2018; Wahlheim et al., 2022). An interesting theoretical question in this case is why the viewer is better off making an anticipatory saccade to the target of the entity’s inferred goal, rather than waiting to find out what the entity does first. One possibility is that predictions, while computationally expensive to generate, function to stabilize the current event model, facilitating information extraction (M. E. Smith & Loschky, 2019; Zacks et al., 2007). Actions in our environment are not random, such that the probability of one action often depends on another. An event model that can capitalize on the regularities of the environment by generating predictions for what will happen next may ease the computational effort associated with information extraction (M. E. Smith & Loschky, 2019). Note that this would seem to be the most active influence of the event model on attentional selection.

These proposed mechanisms point to fertile ground for further work to refine SPECT and elaborate the time course of event model influences on front-end processes. The above mechanisms also provide new hypotheses about how the current event model influences attentional selection, and we have suggested some new ways to test them.

## Conclusion

A key question in vision research is, what influences viewers’ moment-to-moment attentional selection? We highlight a previously largely unexplored top-down influence on attentional selection—namely, the viewer’s event model—their understanding of what is happening now. Borrowing from established fields of literature spanning scene perception, event cognition, and text and discourse comprehension, we describe the event model’s mechanisms for influencing viewers’ attentional selection within scenes. This synthesizes scene perception theories (in the front-end) with theories of viewers’ event models (in the back end). SPECT generates novel testable hypotheses concerning how viewers’ event models influence their attentional selection in dynamic real-world scenes. Importantly, in this update of SPECT, we incorporate the biased competition mechanism of attentional selection, which takes both bottom-up and top-down influences. We also incorporate the ambient-to-focal shift, the fact that the event model resides in working memory (which is well-known to bias attentional selection), and visual search as mechanisms to explain how salient features and the event model compete for selection. We introduce criteria necessary to empirically test the event model hypothesis in the context of narrative film. As such, we believe that SPECT provides a testable and quantifiable theoretical framework for exploring attentional selection in the context of dynamic events. Furthermore, we encourage scene perception researchers to use SPECT, or to propose competing theories, to identify when viewers’ event models affect their attentional selection.

Importantly with respect to this special issue, we see the contributions of SPECT to understanding the event model’s influences on attentional selection as being broadly akin to those made by Mary Peterson regarding top-down effects on object perception. Among other things, she identified how object recognition processes produce top-down influences on figure–ground segmentation, which were previously ignored in the vision sciences because they were thought to be impossible. We see SPECT as similarly providing a theoretical framework that provides vision sciences with a basis for how and when event models affect attentional selection. This theoretical space has largely been ignored till now, because the constituent research areas encompassed by SPECT, namely scene perception, event cognition, and text comprehension, have previously remained siloed. Just as Mary Peterson has shown that “impossible” influences in object perception do in fact occur, we think event models can influence attentional selection in dynamic scenes in ways that can be studied and understood.

## Data Availability

Not applicable

## Appendix: Changes to the SPECT box & arrow model V2 figure from the previous version (Loschky et al., 2018, 2020):

1. Substantive Changes:
  - In “Stimulus”:
    i. “Saliency” has been changed to “Saliency-engendering features.” This is to reflect the fact that saliency is not inherently a stimulus property. Rather, stimulus features engender the perceptual process of determining saliency.
  - In “Back-End”
    i. Downward arrows from “Semantic Memory (Prior Knowledge)” and “Episodic Memory (Stored Event Model)” only touch the “Current Event Model (Episodic Buffer/LT WM)” rather than connecting to specific processes in the “Current Event Model”. This avoids making strong claims about how Semantic Memory and Episodic Memory influence the sub-processes of “Laying the Foundation,” “Mapping incoming info,” and “Shifting.”
2. Changes to ease cognitive processing:
  - The entire figure has been rotated 180 degrees so that “top-down” (feedback) influences are at the top, and “bottom-up” (feed-forward) influences are at the bottom. This is to make the figure conceptually easier to process.
3. Changes only to add clarity, without changing substance:
  - In “Stimulus”:
    i. Arrows from “Saliency-engendering features” and “Medium-specific features” to both “broad” and “narrow” information extraction (“Info extraction”) are explicitly shown. This adds clarity to the previous version of the figure.
    ii. Arrows from “Saliency-engendering features” and “Medium-specific features” to “Attentional Selection” are explicitly shown. This adds greater clarity to the previous version of the figure.
  - In “Front-End”:
    i. The Front-End now has a separate dashed line surrounding it, similar to that for the Back-End, and for the Stimulus. Previously, there was a dashed line around both the Front-End and Back-End, indicating that they are separate from the Stimulus. That dashed line has been removed. These changes simply clarify that the Stimulus, Front-End, and Back-End are each considered separate.
    ii. Boxes around the eye icon, which encompass both “Info extraction” and “Attentional Selection” are now labeled “Fixation” to clarify that both processes are occurring on each fixation. This adds clarity to the previous version of the figure.
  - In “Back-End”:
    i. Downward arrows from the Back-End “Current Event Model (Episodic Buffer/LT WM)” to “Front-End” “Info Extraction” and “Attentional Selection” touch those processes. This is to add visual clarity.
      1. However, the lack of such a downward arrow from the “Current Event Model…” to “Info Extraction Narrow” does not imply that this relationship does not exist. The lack of this arrow is only to avoid visual clutter. It is assumed that the “Current Event Model…” equally influences both “Broad” and “Narrow” information extraction.

## Footnotes

1 We intentionally combine the left and right hemifields here, to capture the entire visual field, while recognizing that each hemisphere of the brain only processes half of that.

2 Note that while SPECT has adopted the three phases from Gernsbacher’s (1990) structure building framework, it has lightly modified certain constructs of it. First, Gernsbacher does not distinguish between long-term and working memory, whereas SPECT does. Second, Gernsbacher uses the term “structures” rather than the specific theoretical constructs of the *current event model*, or *stored event model*, used by SPECT. Third, Gernsbacher articulated these processing phases as operating on the coarse/global scene structure of narratives (e.g., goal-directed actions occurring in a specific time and place within a narrative world). However, SPECT assumes that the three phases operate at both *fine* and *coarse* grain sizes (Kurby & Zacks, 2011).

3 We thank Jonathan Frome for this insight, many years ago.

4 Differential elements are differential event indices (Zwaan & Radvansky, 1998), causal criteria (van den Broek, 1990), and emotions of agents (Gernsbacher et al., 1992) that enable predictions unique to the participants who know the context of the scene. We have identified six primary differential elements based on the event indexing model, the causal inference maker, and the structure building framework:

1. Entities are agents/objects/highly delimited places of interest which are the designated targets of attention.
2. Goals are the purpose or motivations of an agent, typically the target.
3. Temporal operativity indicates whether the event that is leading up to the target prediction is still ongoing.
4. Spatial proximity indicates the closeness of entities in the scene to other entities that can lead to targeted predictions.
5. Enabling Features are any feature (other than spatial proximity and temporal operativity) in the story that enable targeted predictions.
6. Emotions indicate any emotion(s) of the agent(s) relevant for the target predictions.

## Declarations

### Open Practices Statement

All data discussed in this paper is based on summaries of existing published results and analyses.
